# Delaying aging is neuroprotective in Parkinson’s disease: a genetic analysis in *C. elegans* models

**DOI:** 10.1038/npjparkd.2015.22

**Published:** 2015-11-19

**Authors:** Jason F Cooper, Dylan J Dues, Katie K Spielbauer, Emily Machiela, Megan M Senchuk, Jeremy M Van Raamsdonk

**Affiliations:** 1 Center for Neurodegenerative Science, Laboratory of Aging and Neurodegenerative Disease, Van Andel Research Institute, Grand Rapids, MI, USA; 2 Department of Translational Science and Molecular Medicine, Michigan State University, Grand Rapids, MI, USA; 3 Department of Genetics, Michigan State University, East Lansing, MI, USA

## Abstract

Aging is the greatest risk factor for the development of Parkinson’s disease (PD). However, the role of aging in the pathogenesis of PD is not known and it is currently uncertain why the symptoms take many decades to develop when inherited mutations that cause the disease can be present from birth. We hypothesize that there are specific changes that take place during the aging process that make cells susceptible to disease-causing mutations that are well-tolerated at younger ages. If so, then interventions that increase lifespan should be beneficial in the treatment of PD. To test this hypothesis, we used the powerful genetics of *C. elegans,* as this worm has been used extensively in aging research. We crossed transgenic worm models of PD expressing either human mutant α-synuclein (A53T) or LRRK2 (G2019S) with the long-lived insulin-IGF1 receptor mutant, *daf-2*. The *daf-2* mutation increased the lifespan of both PD mutants. The increase in lifespan resulting from the *daf-2* mutation rescued the degeneration of dopamine neurons in both worm models of PD and importantly rescued deficits in dopamine-dependent behaviors including basal slowing, ethanol avoidance, and area-restricted searching. Increasing lifespan through *daf-2* mutation also delayed the formation of small aggregates in a worm model of PD expressing α-synuclein in the body wall muscle and rescued deficits in resistance to different stresses that were present in the PD mutant worms. Overall, this work suggests that slowing down the aging process may provide an effective treatment for PD.

## Introduction

Parkinson’s disease (PD) is the second most common neurodegenerative disease affecting as many as 10 million individuals worldwide. PD is characterized by deficits of movement, including resting tremor, bradykinesia, rigidity, postural instability, and shuffling gait. Patients with PD also exhibit non-motor symptoms that can include dementia, loss of smell, sleep disturbances, constipation, and depression. In the brain, PD is characterized by the degeneration of dopaminergic neurons within the substantia nigra (SN). The SN supplies dopamine to the striatum, thereby having an important role in movement. Brains of patients with PD also exhibit protein aggregates known as Lewy bodies. Although symptomatic treatments are available that can reduce motor deficits, there are currently no neuroprotective therapies available that can delay the progression of the disease.

While the pathogenesis of PD is incompletely understood, a number of genes and environmental factors have been implicated. The first gene to be linked to PD was *SNCA*, which encodes *α*-synuclein (α-syn).^1^ Interestingly, both mutations in the *SNCA* gene and increased copy number of wild-type α-syn lead to PD.^2^ Moreover, α-syn aggregates to form Lewy bodies.^3^ The most commonly mutated gene among PD patients is *LRRK2,* which encodes a Leucine Rich Repeat Kinase.^4,5^ Mutations in *SNCA* and *LRRK2* give rise to an autosomal dominant form of PD and are thought to result from a toxic gain of function.

Aging is the greatest risk factor for the development of PD.^6^ Although the prevalence of PD is 0.3% in the general population, this number increases to 1% of those over 60 and 4% of individuals >80 years of age. Thus, in the inherited forms of PD, patients are symptom free for many decades despite the fact that the disease-causing mutation is present from birth. It is currently uncertain why the disease takes many decades to develop and the role of aging in the disease is poorly understood. We hypothesize that there are changes that take place during normal aging that make neurons susceptible to disease-causing mutations that were well tolerated at younger ages. This hypothesis is supported by the fact that disease onset in animal models of PD is proportional to the lifespan of the organism, indicating that the disease progresses according to biological age and not chronological time. In fact there are a number of changes that take place during normal aging that have been implicated in the pathogenesis of PD. These changes include protein aggregation,^7^ increased oxidative stress,^8^ decreased mitochondrial function,^9^ dysfunction of the proteasome,^10^ and impairment of autophagy.^11^

To test the role of aging in PD, we chose to use *Caenorhabditis elegans* models as these worms offer several advantages for the study of both aging and neurodegenerative disease. The first genes to increase lifespan in any organism were identified in *C. elegans*,^12–14^ and since that time more longevity genes have been identified in *C. elegans* than in any other organism (see GenAge website for lists of genes impacting lifespan^15^). Importantly, genes and interventions that increase lifespan in *C. elegans* have been shown to increase lifespan in other species and be associated with longevity in humans. For example, genes of the insulin-IGF1 signaling pathway that were first shown to increase lifespan in *C. elegans*^12^ have subsequently been shown to increase lifespan in mice^16^ and are associated with longevity in humans.^17^

A number of worm models of PD have been generated through either exposing worms to a neurotoxic chemical such as MPP+ or 6-OHDA or by reproducing the genetic defect present in inherited forms of PD.^18–24^ These worm models have been shown to exhibit multiple phenotypic abnormalities including degeneration of dopamine neurons, decreased levels of dopamine, deficits in dopamine-dependent behaviors, decreased survival, aggregation and alterations in movement.

In this study, we chose to examine the effect of delaying aging in two worm models of PD to ensure that any beneficial outcomes we observe are not model specific. We use a genetic approach to delay aging in *α*-syn and LRRK2 worm models of PD by decreasing insulin-IGF1 signaling with a mutation in the insulin-IGF1 receptor gene *daf-2*. We find that delaying aging rescues multiple deficits present in both worm models of PD including the degeneration of dopamine neurons.

## Results

### Worm models of Parkinson’s disease exhibit phenotypic abnormalities that can be used as outcome measures to assess the effect of delaying aging

Before examining the role of aging in PD, we first sought to determine what phenotypic abnormalities are present in worm models of PD that could be used as outcome measures. We choose to focus on α-syn and LRRK2 as both have been implicated in genetic and sporadic forms of PD. These worm models of PD express either human mutant α*-*syn with the A53T mutation (*Pdat-1:: α-syn(A53T)*, which will be referred to as *α-syn(A53T)*)^20^ or human mutant LRRK2 with the G2019S mutation (*Pdat-1::LRRK2(G2019S),Pdat-1::GFP*, which will be referred to as *LRRK2(G2019S)*)^21^ exclusively in dopamine neurons under the *dat-1* promoter. The LRRK2 worms were generated with the co-injection of a transgene expressing GFP in dopamine neurons (*Pdat-1::GFP*). Accordingly, we have used a strain expressing GFP in dopamine neurons as the control for the LRRK2 mutant, while we have used WT (N2) worms as the control for the α*-*syn mutants.

**Figure 1:**
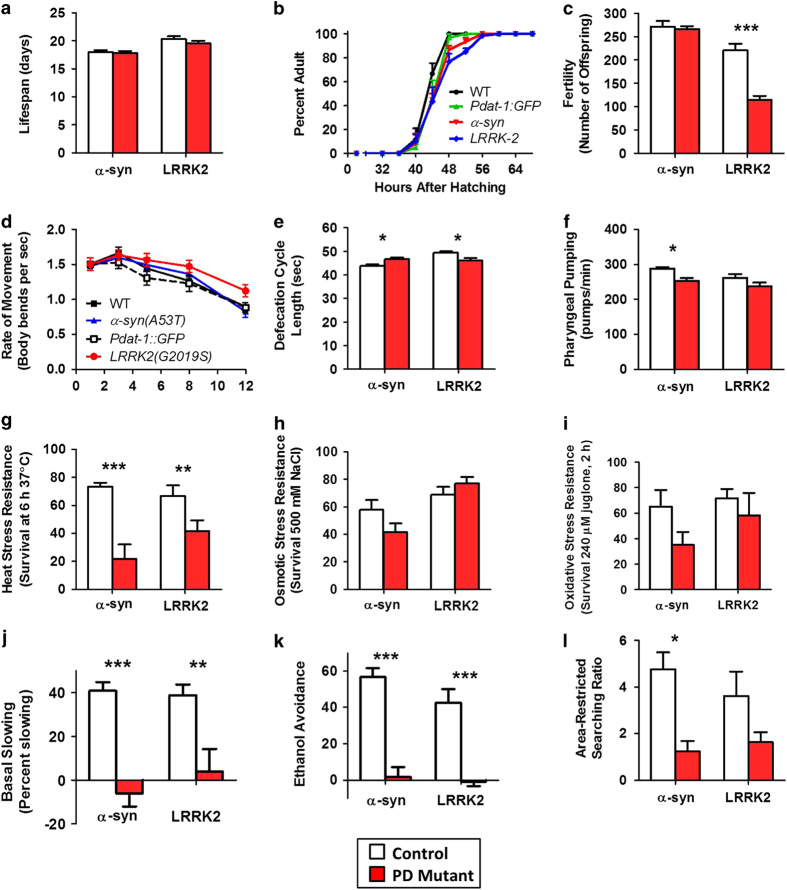
Parkinson’s disease worms exhibit multiple phenotypic abnormalities. In order to determine which phenotypic abnormalities are present in worm models of PD, we examined physiologic rates, stress resistance, and dopamine-dependent behaviors in α-syn (*Pdat-1:: α-syn(A53T*)) and LRRK2 (*Pdat-1::LRRK2(G2019S),Pdat-1::GFP*) worms. Phenotypes assessed included (**a**) lifespan, (**b**) post-embryonic development, (**c**) fertility, (**d**) rate of movement, (**e**) defecation cycle length, (**f**) pharyngeal pumping rate, (**g**) heat stress resistance, (**h**) osmotic stress resistance, (**i**) oxidative stress resistance, (**j**) basal slowing, (**k**) ethanol avoidance, and (**l**) area-restricted searching. We observed significant deficits in fertility, defecation, pharyngeal pumping, heat stress resistance, and all three dopamine-dependent behaviors (basal slowing, ethanol avoidance, and area-restricted searching). White bars indicate control worms, red bars indicate PD mutant worms. The control for α-syn worms was WT. The control for LRRK2 worms was *Pdat-1::GFP*. Error bars indicate SEM. *P<0.05, **P<0.01, ***P<0.001.

Examining the physiologic rates of the PD mutant worms we found that the α*-*syn mutants have normal lifespan, normal post-embryonic development time, normal fertility, normal movement (thrashing rate), a slower rate of defecation, and a decreased pharyngeal pumping rate (Figure 1a–f). α*-*syn mutants also exhibited significantly increased sensitivity to heat stress, normal sensitivity to osmotic stress, and increased sensitivity to oxidative stress (Figure 1g–i). Finally, we examined the ability of α*-*syn worms to perform three dopamine-dependent behaviors: basal slowing,^25^ ethanol avoidance,^26^ and area-restricted searching.^27^ Worms with a mutation in the tyrosine hydroxylase gene *cat-2,* which have depleted levels of dopamine,^28^ exhibit marked deficits in all three behaviors. α*-*syn mutant worms exhibited deficits in all three dopamine-dependent behaviors (Figure 1j–l), suggesting that the expression of mutant α*-*syn in dopamine neurons disrupts dopamine signaling in these worms.

Compared with *Pdat-1::GFP* control worms, LRRK2 mutant worms exhibit a normal lifespan, normal development time, markedly decreased fertility, a trend towards increased thrashing rate, a more rapid rate of defecation, a normal rate of pharyngeal pumping, increased sensitivity to heat stress, normal sensitivity to osmotic stress, and normal sensitivity to oxidative stress (Figure 1a–i). As with α*-*syn mutant worms, LRRK2 mutants show deficits in dopamine-dependent behaviors including an inability to basal slow and lack of ethanol avoidance (Figure 1 j–l). Examination of worms expressing WT α*-*syn or WT LRRK2 revealed that these worms also exhibit some of the phenotypic deficits observed in the mutant strains (Supplementary Figure S1), although the magnitude of the deficit tended to be milder than worms expressing the mutant form of these proteins (Supplementary Table S1).

As the PD mutant worms exhibited differences from WT in pharyngeal pumping, resistance to heat stress and defecation cycle rate, we sought to determine the extent to which these phenotypes might be dopamine dependent. For this purpose we tested *cat-2* tyrosine hydroxylase mutants and found that *cat-2* worms exhibit normal defecation and heat stress resistance but decreased pharyngeal pumping (Supplementary Figure S2). This suggests the possibility that the toxicity of mutant α*-*syn and LRRK2 may cause deficits that are independent of dopamine signaling.

Overall, both α*-*syn and LRRK2 mutant worms exhibit multiple phenotypic abnormalities, which can be used as outcome measures to identify disease modifiers.

### Decreasing insulin/IGF1 signaling increases lifespan in worm models of Parkinson’s disease

Having identified phenotypic differences that can be used as outcome measures to assess the role of aging in PD, we used a genetic approach to determine the effect of delaying aging in the two worm models of PD. We crossed the α*-*syn and LRRK2 transgenic worms to long-lived *daf-2* worms. *daf-2* worms live twice as long as wild-type worms as a result of decreased insulin-IGF1 signaling.^12^

**Figure 2:**
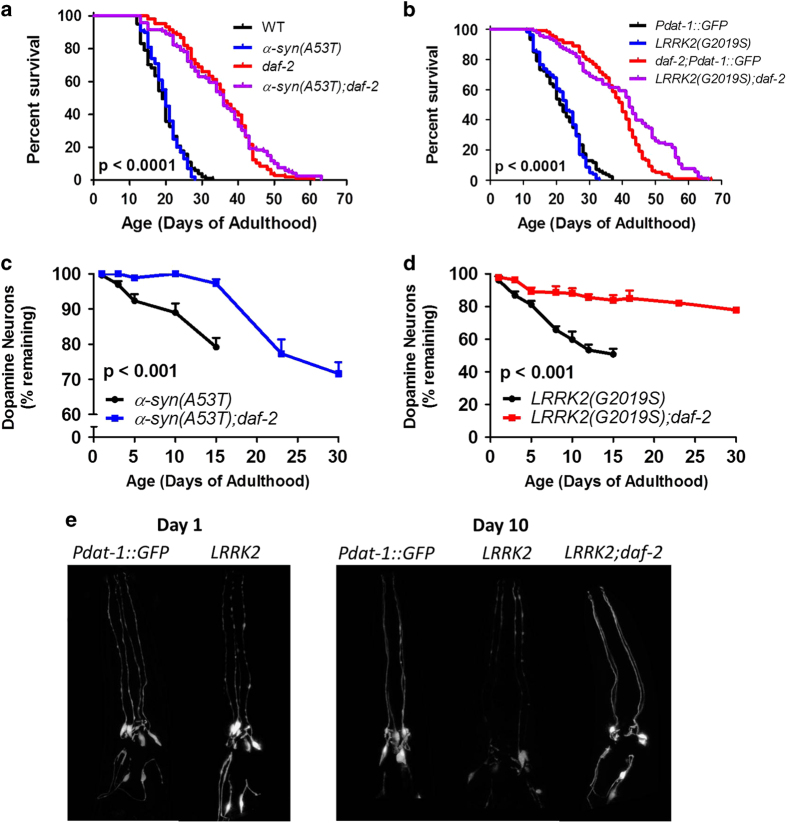
Increasing lifespan reduces the degeneration of dopamine neurons in worm models of Parkinson’s disease. Transgenic worm models of PD expressing human mutant α-synuclein (A53T mutation) or LRRK2 (G2019S mutation) were crossed to long-lived *daf-2* mutants. The *daf-2* mutation increased lifespan in both the α-synuclein (**a**); *Pdat-1::α-syn(A53T)*) and LRRK2 (**b**); *Pdat-1::LRRK2(G2019S),Pdat-1::GFP*) models of PD. To visualize the loss of dopamine neurons, GFP was expressed in dopamine neurons under the *dat-1* dopamine transporter promoter. The anterior six dopamine neurons (2 ADEs and 4 CEPs) were counted throughout the lifespan of worms. (**c**) Increasing lifespan in α-synuclein(A53T) mutant worms decreased the loss of GFP-positive dopamine neurons with age. (**d**) Similarly, the loss of GFP-positive dopamine neurons was markedly reduced in LRRK2(G2019S) mutant worms in the presence of the lifespan-extending mutation in *daf-2.* This indicates that delaying aging is neuroprotective in worm models of PD. (**e**) Representative images of dopamine neurons indicates that LRRK2 worms have a full complement of dopamine neurons on day1 of adulthood. However, the loss of GFP-positive neurons is evident by day 10 of adulthood. This loss is recused by delaying aging with the *daf-2* mutation. Error bars indicate SEM.

Before examining the effect of the *daf-2* mutation on the abnormal phenotypes present in these worm models of PD, we first wanted to ensure that the *daf-2* mutation was able to increase lifespan in the two PD mutants. We found that α*-syn(A53T)* worms live as long as wild-type worms and that the double mutant α*-syn(A53T);daf-2* worms exhibited increased lifespan compared with α*-syn(A53T)* worms, thereby indicating that the *daf-2* mutation had the intended effect of delaying aging in α*-syn(A53T)* worms (Figure 2a). Similarly, we found that *LRRK2(G2019S)* worms have a normal lifespan and that their lifespan is increased by the *daf-2* mutation (Figure 2b). As an additional measure of slowing aging we monitored the accumulation of lipofuscin with age. Lipofuscin is a product of oxidative damage that accumulates with age and has been used as a marker of aging. We found that in both *α-syn(A53T);daf-2* worms and *LRRK2;daf-2* worms there was a markedly slower accumulation of lipofuscin than in *α-syn(A53T)* and *LRRK2* mutants (Supplementary Figure S3). This indicates that the *daf-2* mutation is effective in delaying aging in both PD mutants.

### Delaying aging increases the survival of dopamine neurons in worm models of Parkinson’s disease

Having shown that the *daf-2* mutation could delay aging in both PD mutants, we next sought to determine whether delaying aging would have a beneficial effect on the abnormal phenotypes present in these strains. As PD is characterized by the selective loss of dopamine neurons in the SN, we first examined the degeneration of dopamine neurons with increasing age. As has been previously reported,^21,29^ we found that there is a slow degeneration of dopamine neurons with increasing age in wild-type worms that is accelerated by expression of α-syn(A53T) or LRRK2(G2019S; Supplementary Figure S5). Comparing α*-syn(A53T)* worms and α*-syn(A53T);daf-2* worms, we found that the *daf-2* mutation resulted in a significantly slower rate of dopamine neuron loss with age (Figure 2c). Similarly, the degeneration of dopamine neurons was markedly less in *LRRK2(G2019S);daf-2* worms than in *LRRK2(G2019S)* worms (Figure 2d,e). In fact, at day 30, when all of the *LRRK2(G2019S)* worms have died, *LRRK2(G2019S);daf-2* worms have a similar number of dopamine neurons to *LRRK2(G2019S)* worms on day 5. Combined this indicates that delaying aging can markedly slow the degeneration of dopamine neurons in worm models of PD.

We also examined the dendrites of dopamine neurons for signs of degeneration prior to the loss of the neuronal cell body. We quantified dendritic blebs (see Supplementary Figure S6 for image) and found that these blebs increased with age and were found more frequently in *LRRK2(G2019S)* PD mutants (Supplementary Figure S6).

### Delaying aging improves dopamine dependent behaviors in worm models of Parkinson’s disease

**Figure 3:**
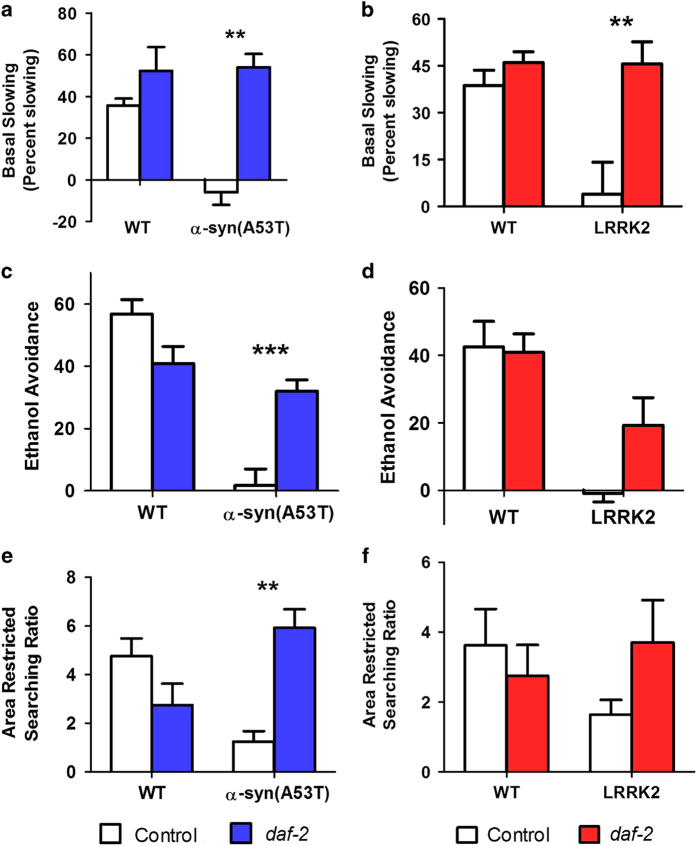
Delaying aging rescues deficits in dopamine-dependent behaviors in worm models of Parkinson’s disease. (**a** and **b**) α-syn and LRRK2 PD mutants exhibit a deficit in basal slowing that is completely rescued by delaying aging. (**c** and **d**) Similarly, increasing lifespan through the *daf-2* mutation partially restores the ethanol avoidance response in α-syn and LRRK2 PD mutants. (**e** and **f**) Although the *daf-2* mutant worms exhibit a trend toward decreased area-restricted searching, the *daf-2* mutation increases searching in both α-syn and LRRK2 PD mutants. White bars indicate control worms, red/blue bars indicate PD mutant worms. α-syn=*Pdat-1:: α-syn(A53T*), LRRK2=*Pdat-1::LRRK2(G2019S),Pdat-1::GFP*. The control for α-syn worms was WT. The control for LRRK2 worms was *Pdat-1::GFP*. Error bars indicate SEM. **P<0.01, ***P<0.001.

Having shown that delaying aging through the *daf-2* mutation is neuroprotective in worm models of PD, we next sought to determine the extent to which neuronal function is preserved. To do this, we examined the ability of the *daf-2* mutation to rescue deficits in dopamine-dependent behaviors. We found that while both α-syn(A53T) or LRRK2(G2019S) mutants exhibit little or no basal slowing, α*-syn(A53T);daf-2* and *LRRK2(G2019S);daf-2* demonstrate basal slowing that is indistinguishable from wild-type (Figure 3a and b). Similarly, despite the fact that neither PD mutant strain showed any degree of ethanol avoidance, in both cases the *daf-2* mutation partially restored function towards wild-type (Figure 3c and d). Finally, in the area-restricted searching assay, we found that both α*-syn(A53T);daf-2* and *LRRK2(G2019S);daf-2* mutants exhibited increased searching compared with α*-syn(A53T)* and *LRRK2(G2019S)* single mutants, respectively (Figure 3e and f). Thus, in addition to preserving the number of GFP-positive dopamine neurons in PD mutant worms, delaying aging through the *daf-2* mutation also has a functional impact in preventing the loss of dopamine-dependent behaviors.

We also examined the effect of the *daf-2* mutation on physiologic rates in the α*-*syn and LRRK2 PD mutant worms. We found that delaying aging through mutation of *daf-2* did not improve fertility (Supplementary Figure S7a,b), pharyngeal pumping rate (Supplementary Figure S7c,d), or thrashing rate (Supplementary Figure S7e,f) in either PD mutant. Interestingly, the expression of LRRK2(G2019S) in dopamine neurons significantly increased the rate of movement of *daf-2* mutant worms (Supplementary Figure S7f).

### Delaying aging rescues deficits in resistance to stress in worm models of Parkinson’s disease

**Figure 4:**
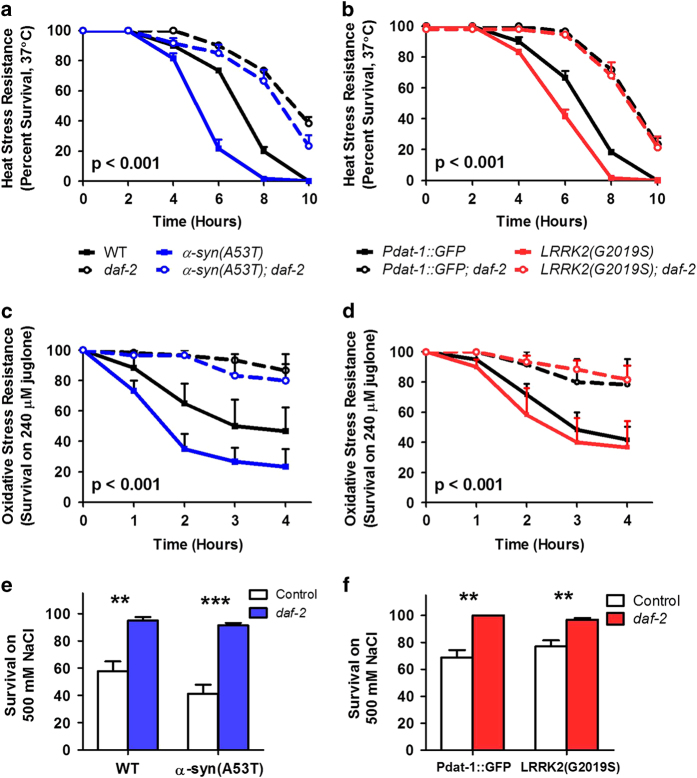
Delaying aging through mutation of *daf-2* rescues decreased stress resistance in Parkinson’s disease worms. (**a**) α-syn(A53T) worms have increased sensitivity to heat stress that is rescued by the *daf-2* mutation. (**b**) Similarly, LRRK2(G2019S) worms are more sensitive to heat stress than control and this deficit is ameliorated by *daf-2* mutation. Increasing lifespan through *daf-2* mutation also improves resistance to oxidative stress (**c** and **d**) and osmotic stress (**e** and **f**). Error bars indicate SEM. *P*-values indicate difference between PD mutant and PD; *daf-2* double mutant. **P<0.01, ***P<0.001.

Increasing lifespan through mutations in *daf-2* has been shown to increase resistance to a variety of stresses including heat stress,^30^ oxidative stress,^31^ and osmotic stress.^32^ As both PD mutants exhibited increased sensitivity to different types of stress, we next determined the extent to which increasing lifespan through the *daf-2* mutation could rescue the deficits in stress resistance in α*-*syn and LRRK2 transgenic worms. Both α*-*syn and LRRK2 mutants exhibit increased sensitivity to heat stress (Figure 4a and b). In both cases the deficit was completely rescued in the *daf-2* double mutant (Figure 4a and b). Similarly, the increased sensitivity to oxidative stress that is present in *α-syn(A53T)* mutant worms is ameliorated by the *daf-2* mutation (Figure 4c). Finally, we found that delaying aging through *daf-2* mutation also improved resistance to oxidative stress in *LRRK2(G2019S)* mutant worms and osmotic stress resistance in both α*-*syn and LRRK2 PD worm models (Figure 4d and f).

### Delaying aging decreases the formation of small α*-*synuclein aggregates

**Figure 5:**
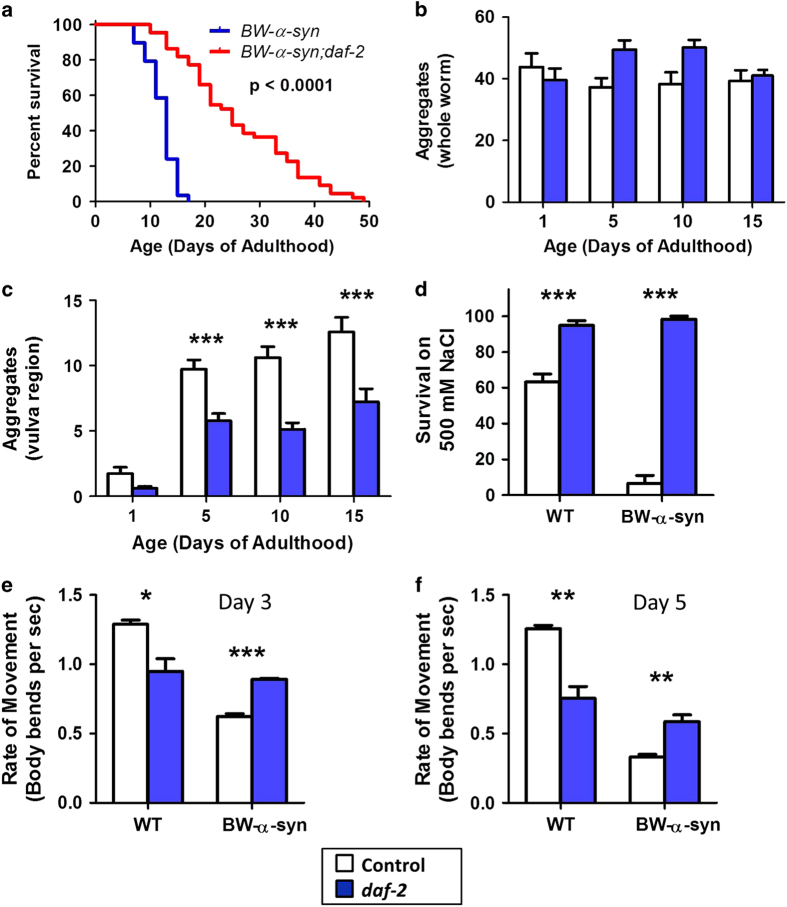
Delaying aging through mutation of *daf-2* is beneficial in a worm model of Parkinson’s disease expressing α-synuclein in body wall muscle. (**a**) The *daf-2* mutation increases lifespan in *Punc-54::α-syn(WT):YFP* (*BW-α-syn*) worms. (**b**) Aggregates of α-synuclein were monitored by expressing wild-type α-synuclein linked to YFP in body wall muscle under the *unc-54* promoter. Whole-worm counts of large aggregates shows no difference between control and *daf-2.* (**c**) Counting smaller aggregates in a defined region surrounding the vulva reveals decreased aggregation in the *daf-2* double mutant worms. (**d**) *BW-α-syn* worms show increased sensitivity to osmotic stress, which is completely rescued by the *daf-2* mutation. (**e** and **f**) *BW-α-syn* also exhibit decreased movement. Despite the fact that the *daf-2* mutation decreases movement, *BW-α-syn;daf-2* worms exhibited increased movement compared with *BW-α-syn* worms and their movement was indistinguishable from *daf-2* mutants alone. (**e**) Day 3 of adulthood. (**f**) Day 5 of adulthood. Error bars indicate SEM. *P<0.05, **P<0.01, ***P<0.001.

As PD is characterized by the formation of α*-*syn aggregates called Lewy bodies, we next sought to determine whether delaying aging would also decrease aggregation. To be able to visualize aggregation in live worms, we utilized a worm model of PD in which wild-type α*-*syn is linked to yellow fluorescent protein (YFP) for easy visualization and expressed in body wall muscle under the *unc-54* promoter (*Punc-54:: α-syn(WT):YFP*), which will be referred to as BW-α-syn worms. These worms have previously been shown to exhibit aggregation that increases with age.^24^ We crossed the BW-α-syn worms with *daf-2* mutants in order to delay aging (Figure 5a) and examined the formation of aggregates with increasing age. Unlike worms expressing a mutant length of polyglutamine linked to YFP under the same *unc-54* promoter, which exhibit large aggregates, BW-α-syn have smaller aggregates of varying sizes (Supplementary Figure S8). To compensate for this heterogeneity, we used two different paradigms: (1) we quantified aggregates that could be seen under low magnification (dissecting fluorescent microscope) in whole worm images and (2) we quantified aggregates in a defined area surrounding the vulva under high magnification (compound fluorescent microscope). We found that while the *daf-2* mutation had little impact on the number of aggregates that could be visualized in whole worms (Figure 5b), it resulted in a significant decrease in smaller aggregates surrounding the vulva (Figure 5c).

As BW-α-syn PD mutant worms exhibit deficits in osmotic stress resistance and movement, we next examined whether *daf-2* mutation would be able to rescue these deficits. We found that the markedly decreased survival of BW-α-syn worms under osmotic stress (500 mM NaCl) was completely rescued in *BW-α-syn*;*daf-2* double mutants such that the double mutants survived better than wild-type (Figure 5d). Similarly, we found that BW-α-syn worms have decreased movement in a thrashing assay (Figure 5e and f). Despite the fact that *daf-2* mutation results in decreased movement on a wild-type background, the *daf-2* mutation significantly improved movement in BW-α-syn mutant worms (Figure 5e and f). In fact, the rate of movement in *BW-α-syn*;*daf-2* worms and *daf-2* worms was indistinguishable.

### Decreasing levels of FOXO transcription factor DAF-16 prevents the beneficial effects of *daf-2* mutation in LRRK2 worm model of Parkinson’s disease

The insulin-IGF1 receptor encoded by the *daf-2* gene modulates gene expression by controlling the nuclear localization of the FOXO transcription factor DAF-16. Accordingly, the ability of the *daf-2* mutation to increase lifespan is entirely dependent on DAF-16.^12^ To determine whether signaling through DAF-16 is required for the beneficial effect we observe in worm models of PD, we used RNAi to knockdown DAF-16 levels in *LRRK2(G2019S);daf-2* worms. We found that *daf-16* RNAi was effective at knocking down DAF-16:GFP expression in a Pdaf-16:daf-16:GFP reporter strain (Supplementary Figure S9a). However, we noticed that there was still some residual DAF-16:GFP expression in neurons, which is consistent with previous observations that RNAi is less efficient in neurons.

**Figure 6:**
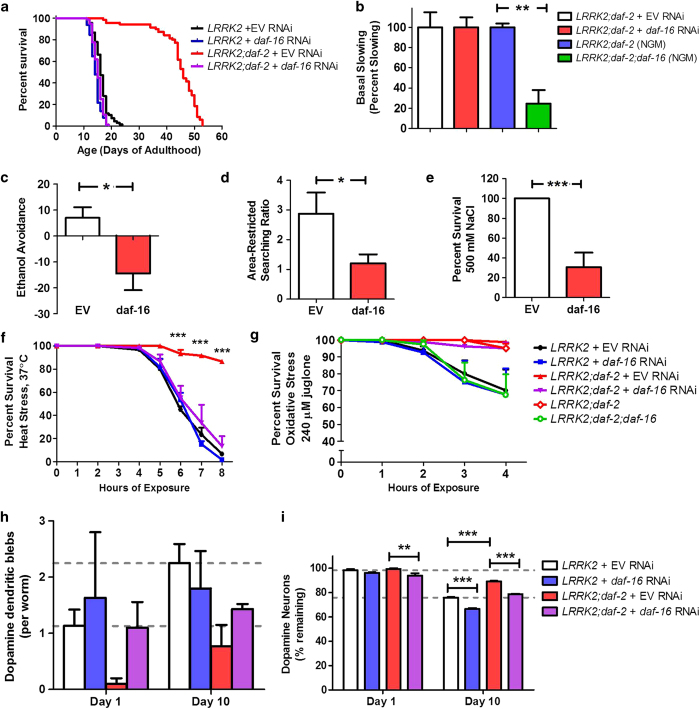
Beneficial effects of *daf-2* mutation in LRRK2(G2019S) worm model of Parkinson’s disease are mediated by DAF-16. To test the role of canonical DAF-2 signaling through the DAF-16 transcription factor in mediating the beneficial effect of *daf-2* mutation in worm models of Parkinson’s disease, we treated *LRRK2(G2019S);daf-2* worms with *daf-16* RNAi to determine the extent to which phenotypic improvements caused by the *daf-2* mutation would be lost. Decreasing *daf-16* expression by RNAi completely abolished the increased lifespan in *LRRK2(G2019S);daf-2* worms (**a**), and reduced the beneficial effect of *daf-2* mutation on ethanol avoidance (**c**), area-restricted searching (**d**), resistance to osmotic stress (**e**), heat stress resistance (**f**), dopamine dendritic blebs (**h**) and loss of dopamine neurons (**i**). In contrast, *daf-16* RNAi treated *LRRK2(G2019S);daf-2* worms still showed a benefit compared with *LRRK2(G2019S)* worms in basal slowing (**b**), and oxidative stress resistance (**g**). To test whether this was a result of residual *daf-16* expression in neurons, we generated *LRRK2;daf-2;daf-16* triple mutant worms. Deletion of *daf-16* abolished the beneficial effect of the *daf-2* mutation on both basal slowing and oxidative stress resistance in *LRRK2;daf-2* worms (**b** and **g**). Thus, the beneficial effect of *daf-2* mutation is completely mediated by DAF-16. Error bars indicate SEM. *P<0.05, **P<0.01, ***P<0.001.

To assess the impact of *daf-16* RNAi on the ability of the *daf-2* mutation to increase longevity in *LRRK2(G2019S)* worms, we examined lifespan and lipofuscin accumulation. We found that *daf-16* RNAi completely abolished the increased lifespan resulting from *daf-2* mutation (Figure 6a). Similarly, lipofuscin accumulation was greater in *daf-16* RNAi treated worms suggesting that the ability of the *daf-2* mutation to delay aging was inhibited by our *daf-16* RNAi treatment paradigm (Supplementary Figure S9b).

Having shown that *daf-16* RNAi could prevent the beneficial effect of *daf-2* mutation on lifespan, we next sought to determine if *daf-16* RNAi would also prevent the amelioration of phenotypic abnormalities observed in *LRRK2(G2019S);daf-2* PD mutants. We found that *daf-16* RNAi reduced the beneficial effect of *daf-2* mutation on ethanol avoidance, area-restricted searching, osmotic stress resistance, heat stress resistance, dendritic blebs, and loss of dopamine neurons (Figure 6). In contrast, *daf-16* RNAi did not worsen basal slowing or oxidative stress resistance in *LRRK2(G2019S);daf-2* worms (Figure 6b and g). To determine whether this might be due to the residual DAF-16 expression that we observed in neurons, we generated *LRRK2(G2019S);daf-2;daf-16* triple mutant worms. We found that the *daf-16* mutation prevented the improvement in basal slowing and completely abolished the improved resistance to oxidative stress in *LRRK2(G2019S);daf-2* worms (Figure 6b and g). Overall, this indicates that the beneficial effect of *daf-2* mutation in worm models of PD is mediated by DAF-16.

## Discussion

### Aging and Parkinson’s disease

Aging is the greatest risk factor for PD.^33^ Advancing age not only increases the likelihood that an individual will develop PD,^6^ but those that develop PD at a later age have a more severe disease course in terms of motor impairment, levodopa responsiveness, gait and postural deficits, and dementia.^34^ In fact, there are many commonalities between PD and the normal process of aging (for a comprehensive summary see Table 1 in ref. 35). The pattern of selective cell loss in the brain is similar between PD and aging.^33^ Accumulation of α-syn protein occurs with advancing age and in PD.^36^ Both aging and PD are believed to be caused by a complex combination of genes and environmental factors, with relatively few genes having major effects.^37^ In addition, many of the changes that take place during normal aging, have been implicated in the pathogenesis of PD including increased aggregation,^7^ increased oxidative stress,^8^ decreased mitochondrial function,^9^ dysfunction of the proteasome,^10^ and impairment of autophagy.^11^ On the basis of the large number of similarities between aging and PD, it has been proposed that PD may result from aging.^35^ If so, then delaying aging may be an effective strategy to treat PD. Our results provide a proof of principle that delaying aging can be neuroprotective in PD.

### Worm models of Parkinson’s exhibit loss of dopamine neurons and deficits in dopamine-dependent behaviors

In the characterization of α-syn and LRRK2 mutant worms, we were able to reproduce originally reported phenotypes in these strains^20,21^ and to identify novel deficits. All of the phenotypes present in these strains result from the expression of the mutant protein in the eight dopamine neurons present in the worm (it is possible that the *dat-1* promoter drives low level of expression in other tissues). This comprises a small fraction of the 302 neurons and 959 cells that are present in the worm. The loss of dopamine neurons in the SN is a characteristic feature of PD. It has also been shown that the number of dopamine neurons declines with age in unaffected individuals albeit at a decreased rate compared with PD. Similarly, we found that GFP-positive dopamine neurons were lost with increasing age in wild-type worms and the rate of loss is accelerated in both PD mutants. We also observed deficits in multiple dopamine-dependent behaviors including basal slowing, ethanol avoidance, and area-restricted searching. We have observed similar deficits in *cat-2* mutant worms that have a mutation in the gene encoding tyrosine hydroxylase. As tyrosine hydroxylase is required for the synthesis of dopamine, the *cat-2* worms have decreased levels of dopamine. Similarly, dopamine levels have previously been shown to be decreased in both the α*-*syn and LRRK2 worms models used in our study.^20,21^

Our results show an increased sensitivity to heat stress in both α-syn and LRRK2 mutants, as well as an increased sensitivity to oxidative stress in α-syn mutant worms. This is surprising given that the expression is limited to the eight dopamine neurons. Nonetheless, this is consistent with studies on aging showing that specific neurons can modulate survival in response to temperature.^38^ We also observe a marked decreased in fertility in LRRK2 mutants. Previous studies have shown that exogenous dopamine inhibits egg laying.^39^ As LRRK2(G2019S) mutants have decreased levels of dopamine,^21^ perhaps endogenous, and exogenous dopamine have different effects on egg laying.

The fact that α-syn and LRRK2 mutants exhibit deficits in defecation cycle length and heat stress resistance that are not observed in *cat-2* mutants, which have diminished levels of dopamine, suggests the possibility that these may be dopamine-independent deficits resulting from α-syn and LRRK2 toxicity. There are multiple possibilities that could explain the additional deficits present in the PD worms. First, the expression of a toxic protein such as LRRK2 or α-synuclein could disrupt a function of the CEP, ADE, or PDE neurons that is not mediated by dopamine. Second, the expression of a toxic protein such as LRRK2 or α-synuclein could cause the dopamine neurons to release chemokines that negatively influence neighboring cells. Third, at least in the case of the α-synuclein mutants, there may be prion-like transfer to other non-dopamine neurons that may contribute to these phenotypes.

### Delaying aging by mutation of *daf-2* is beneficial in worm models of Parkinson’s disease

In this study, we focused on the insulin-IGF1 signaling pathway as a means of delaying aging in worm models of PD. The *daf-2* gene encodes the insulin-IGF1 receptor and mutations in this gene have been shown to more than double lifespan in the worm.^12^ After the insulin-IGF1 signaling pathway was shown to increase lifespan in *C. elegans*, genes in this same pathway were found to increase lifespan in yeast, flies and mice, thereby indicating conservation across species.^40^ Importantly, genes in this same pathway have been shown to be associated with longevity in humans.^17^

We found that delaying aging through the mutation of *daf-2* is neuroprotective in two different genetic models of PD. In fact, *LRRK2(G2019S);daf-2* double mutants have more dopamine neurons remaining at day 30 (10 days past the average lifespan of *LRRK2(G2019S)* and WT worms) than *LRRK2(G2019S)* mutants have on day 8 of adulthood. Although the rate of neuronal loss in *α-syn(A53T)* transgenic worms is decreased compared with *LRRK2(G2019S)* worms, there is still a significant rescue imparted by the *daf-2* mutation. We also found that delaying aging rescued deficits in dopamine-dependent behaviors and abolished the increased sensitivity to stress observed in the PD mutants. We did not observe any beneficial effect on the reduced fertility present in *LRRK2(G2019S)* mutants, but perhaps this is due to the fact that the *daf-2* mutation decreases brood size in wild-type worms. While we were completing our study, the Caldwell group showed a beneficial effect of the *daf-2* mutation in a different α-syn model of PD.^41^ In their study, they utilized a transgenic worm expressing wild-type α-syn and found that a mutation in *daf-2* could rescue dopaminergic degeneration and decrease aggregation in body wall muscle. The fact that two independent experiments in different labs with different models both show a beneficial effect of delaying aging in worm models of PD provides strong support that this may be an effective target for treating PD. In addition, a screen for modifiers of α-syn aggregation identified multiple aging genes that were able to suppress aggregate formation.^24^

Further support for this strategy comes from experiments showing a beneficial effect of interventions that increase lifespan in chemically induced rodent models of PD. Resveratrol, a potent enhancer of the histone deacetylase Sirtuin 1 activity that has been shown to increase lifespan in worms, flies, and mice,^42^ has been shown to be protective in the 6-OHDA rat model of PD.^43^ Rapamycin, an inhibitor of mTOR signaling that increases lifespan in mice,^44^ was shown to be protective in the MPTP mouse model of PD.^45^ Dietary restriction, which has been shown to increase lifespan in yeast, worms, flies, mice, and monkeys,^46^ improved survival of dopamine neurons in MPTP-treated rodents^47^ and resulted in better maintenance of motor activity in MPTP-treated primates.^48^ Finally, treatment with the anti-diabetic drug metformin has been shown to increase lifespan in worms and mice^49,50^ and has been found to improve neuronal survival, motor function, and behavior following MPTP treatment in mice.^51^ Overall, the fact that multiple interventions that increase lifespan exhibit a beneficial effect in PD suggests that targeting the aging process may be an effective strategy for treating PD.

### Targeting aging in mouse models of PD and PD patients

Whereas there is a single insulin-IGF1 receptor in *C. elegans,* in mice and humans there are separate receptors for insulin and IGF1. Nonetheless, evidence from a number of mouse models indicates that decreasing either insulin or IGF1 signaling can lead to increased lifespan in mice as well. Mice bearing a heterozygous deletion in the IGF1 receptor gene *Igf1r* are long lived,^16^ as are mice with a homozygous deletion in the insulin receptor that is limited to adipose tissue.^52^ Mice with a heterozygous deletion in the insulin receptor substrate 2 gene (*Irs2*), either throughout the body or limited to just the brain, are also long lived.^53^ Long-lived Snell dwarf mice, Ames dwarf mice, and growth hormone receptor mutants have all been shown to have decreased levels of IGF1.^54–56^ Finally, mice expressing decreased levels of IGF1 have been shown to have increased maximum lifespan.^57^

In translating the results from the present study to mice and eventually humans, it will be essential to determine in which tissue(s) insulin-IGF1 signaling must be decreased in order to have a beneficial effect in PD. Decreasing insulin or IGF1 signaling in mammals may be complicated by potential detrimental side effects. For example, loss of insulin signaling in the pancreas could lead to diabetes. However, if the neuroprotective effect of the *daf-2* mutation is cell autonomous, then it may be possible to reduce insulin signaling specifically in dopamine neurons, thereby avoiding or minimizing the possibility of side effects. As it has previously been shown that decreasing insulin-IGF1 signaling in neurons is required for the long lifespan of *daf-2* mutants,^58^ it is possible that reducing insulin-IGF1 signaling in neurons will be sufficient to protect the neurons from the toxic effects of mutant α-syn or *LRRK2.*

One previous study examined the effect of decreasing IGF1 signaling in a mouse model of PD. Heterozygous *Igf1r*+/− mice were treated with MPTP and were found to have a larger lesion than WT controls.^59^ Although this result appears to contradict the results from our study in *C. elegans*, it should be noted that the *Igf1r*+/− mice only have a mild increase in lifespan and the MPTP model involves an acute exposure to highly toxic chemical. This model recapitulates the neuronal loss present in PD, but does not reproduce the pathogenesis of the disease. Thus, it is possible that delaying aging through decreasing insulin or IGF1 signaling may be beneficial in a genetic mouse model of PD that more accurately reproduces the pathogenic mechanisms underlying PD. Support for this conclusion comes from the fact that decreasing insulin or IGF1 signaling has previously been shown to be beneficial in genetic mouse models of other neurodegenerative diseases including Alzheimer’s disease^60^ and Huntington’s disease.^61^

## Conclusions

Overall, we show that delaying aging through decreasing insulin-IGF1 signaling is neuroprotective in worm models of PD. Future studies will need to validate these results in genetic mouse models of PD. As decreasing insulin or IGF1 signaling may have detrimental effects in humans it may be necessary to target specific tissues and tightly control the degree to which signaling is knocked down. As an alternative approach, it may be fruitful to explore other pathways of lifespan extension, which are less likely to induce side effects.

## Materials and methods

### *C. elegans* strains and maintenance

The following strains were used in this study:

N2(WT),

BY250 vtIs7[pDAT::GFP(pRB490)],

CB1370 daf-2(e1370) III,

JVR209 cat-2(e1112),

JVR120 daf-16(mu86) I,

TJ356 zIs356[Pdaf-16::daf-16:GFP],

JVR103 Pdat-1:: α-synuclein[WT],

JVR107 Pdat-1::α-synuclein[A53T],

JVR203 Pdat-1::α-synuclein[A53T]; vtIs7[pDAT::GFP(pRB490)],

JVR206 daf-2(e1370) III; vtIs7[pDAT::GFP(pRB490)],

JVR205 daf-2(e1370) III; Pdat-1:: α-synuclein[A53T],

JVR210 daf-2(e1370) III; Pdat-1:: α-synuclein[A53T]; vtIs7[pDAT::GFP(pRB490)],

JVR105 cwrIs730 [Pdat-1::GFP, lin-15(+)],

JVR189 daf-2(e1370) III; cwrIs730 [Pdat-1::GFP, lin-15(+)],

JVR104 cwrIs856 [Pdat-1::GFP, Pdat-1::LRRK2(WT), lin-15(+)],

JVR168 cwrIs722 [Pdat-1::GFP, Pdat-1::LRRK2(WT), lin-15(+)],

JVR202 daf-2(e1370) III; cwrIs722 [Pdat-1::GFP, Pdat-1::LRRK2(WT), lin-15(+)],

NL5901 pkIs2386[punc-54:: α-synuclein::YFP+unc-119(+)],

JVR192 daf-2(e1370) III; pkIs2386[punc-54:: α-synuclein::YFP+unc-119(+)],

JVR323 daf-16(mu86) I; daf-2(e1370) III; cwrIs722 [Pdat-1::GFP, Pdat-1::LRRK2(WT), lin-15(+)].

All strains were maintained at 16 °C to prevent dauer formation in the *daf-2* mutant strains. Worms were shifted to 20 °C on day 1 of adulthood for the completion of phenotyping assays. Strains were outcrossed to our N2 strain six times to obtain a uniform strain background.

### Generation of double mutants

Double mutants were generated by standard techniques. In most cases, N2 males were crossed to GFP/YFP positive worms (e.g., *Pdat-1::GFP*). Fluorescent males from the progeny were crossed to a deletion mutant (e.g., *daf-2*). The resulting fluorescent hermaphrodites (e.g., *daf-2/+; Pdat-1::GFP/+*) were selfed. Eight fluorescent progeny was then singled and the genotype determined by PCR for deletion mutants or sequencing for point mutants. In the case of *daf-2* crosses, eggs and L1 worms from the second cross were transferred to 25 °C. After 2–3 days, dauers were transferred to a new plate. After an additional day at 25 °C, worms that were still dauer were recovered at 16 °C, singled onto individual plates and the genotype of the worm was confirmed by sequencing. Homozygosity of fluorescent transgenes was determined by counting 30 worms in three consecutive generations and observing 100% fluorescence.

### Blinding of experimenters and replicates

All studies were completed such that the experimenter was blind to the genotype of the worms. Strains were given letter codes by another member of the laboratory and the code was not broken until all of the replicates for a particular assay were completed. For all assays, we completed a minimum of three biological replicates per strain.

### RNA interference

For RNAi experiments, individual colonies from freshly streaked LB-Tetracycline-Ampicillin plates were grown for 10–12 h in LB media containing 50 μg/ml carbenicillin. Bacteria was concentrated fivefold and seeded onto NGM plates containing 1 μg/ml IPTG and 50 μg/ml carbenicillin. After 2 days of induction, L4 worms were transferred to RNAi plates. The next day gravid adults were transferred to a new plate. These adults were removed the next day and their progeny were used for experiments.

### Physiologic rates

#### Lifespan

Lifespan was measured on plates containing 25 μM 5-fluoro-2′-deoxyuridine (FUdR) to limit the growth of progeny. This concentration was shown to have minimal effects on lifespan.^62^ As this concentration of FUdR does not completely prevent the development of progeny to adulthood in the first generation, worms were transferred to fresh plates after 4 days. Thereafter, worms were transferred to fresh plates weekly. Viability was assessed every 2 days by gentle prodding with a platinum pick. Worms that either had internal hatching of progeny or expulsion of internal organs were not counted as deaths.

#### Postembryonic development time

Post-embryonic development (PED) time was measured by transferring eggs to an NGM plate. After 3 h, newly hatched L1 worms (20 worms per replicate) were transferred to a new NGM plate. The time from hatching to the L4-adult transition was measured as the PED time.

#### Fertility

Brood size was measured by placing individual worms at the L4 stage onto NGM plates followed by daily transfers to new plates. The resulting progeny was allowed to develop to adulthood before counting (5 worms per replicate).

#### Rate of movement

Rate of movement was assessed by measuring thrashing in liquid using video-tracking and computer analysis. Approximately 50 worms were placed in M9 buffer (22 mM _KH2PO4_, 34 mM _K2HPO4_, 86 mM NaCl, 1 mM MgSO_4_) on a clean NGM plate. Videos were taken with an Allied Vision Tech Stingray F-145 B Firewire Camera (Allied Vision, Exton, PA, USA) at 1024×768 resolution, 8-bit using the MATLAB image acquisition toolbox. Analysis was performed using wrMTrck plugin for ImageJ (publically available at http://www.phage.dk/plugins).

#### Defecation rate

Defecation cycle length was determined by measuring the time between two consecutive pBoc contractions in day-1 adult worms with at least 5 worms per replicate. To minimize the effects of laboratory temperature, defecation was measured on water filled chambers that had been incubated at 20 °C and the lids of the plates containing the worms were not removed.

#### Pharyngeal pumping rate

Pharyngeal pumping rate was assessed by video-recording followed by manual counting in day-1 adult worms with 10 worms per replicate. For each measurement worms were placed on an OP50 bacteria-seeded NGM assay plate and recorded for 30 s. Recordings taken at 15 frames per second were replayed at half speed for manual counting.

### Stress resistance assays: Heat stress, Oxidative stress, Osmotic stress

Sensitivity to heat stress was determined by assessing survival of young adult worms incubated at 35 °C. Survival was assessed hourly. Sensitivity to oxidative stress was determined through multiple paradigms. Sensitivity to acute oxidative stress was assessed through exposure to increasing concentrations of juglone (Sigma, St. Louis, MO, USA). Young adult worms were transferred to freshly prepared NGM plates containing 240 μM juglone, and survival was assessed hourly. Sensitivity to chronic oxidative stress was determined through exposure to plates containing 2 mM paraquat (methyl viologen, Sigma) beginning at day-1 of adulthood. FUdR (100 μM) was added to these plates to prevent internal hatching of progeny. Survival was monitored daily until death. Sensitivity to osmotic stress was determined by transferring young adult worms to NGM plates containing increased concentrations of NaCl (450–500 mM). The survival of worms was determined after 48 h. All stress assays were completed on day-1 of adulthood and included a minimum of 20 worms per replicate.

### Dopamine-dependent behaviors

#### Basal slowing

Approximately 50 worms at day 3 of adulthood were washed in M9 buffer to remove any residual bacteria. The cleaned worms were then transferred to either unseeded NGM plates or NGM plates seeded with OP50 bacteria covering the entire plate. After 5 min, videos of the entire plate were recorded for 1 min with an Allied Vision Tech Stingray F-504 B Firewire Camera and a Navitar Zoom 7000 lens (Navitar, Tokyo, Japan) using and the MATLAB image acquisition tool. Recordings were processed using the wrMTrck plugin for ImageJ. Basal slowing was calculated as the difference in rate of movement on food versus off food divided by the rate of movement off food.

#### Ethanol avoidance assay

Day-1 adult worms were transferred to assay plates, which are divided into four quadrants: two quadrants seeded with 50 μl ethanol and the others without. Worms are placed in the center of the assay plate and allowed to move for 30 min at which point the entire plate is imaged and the worms are scored for their quadrant of preference. Ethanol avoidance is calculated as ((number of worms in control quadrants)−(number of worms in ethanol quadrants))/ total number of worms.

#### Area-restricted searching

Twenty well-fed day-1 adult worms were placed on a clean 60 mm NGM assay plate. One-minute-long video-recordings were taken at 5 min and 30 min after transfer to the assay plate. The tracks of individual worms are then analyzed for the number of turns exceeding 90° are counted. The area-restricted searching ratio is calculated as the number of turns per worm at 5 min divided by the number of turns per worm at 30 min. A minimum of three replicates per strain were completed.

#### Lipofuscin measurements

Synchronized worms at either day 6 or day 9 of adulthood were plated on NGM plates and aged to the appropriate time point. Worms were then transferred to a clean 60mm NGM plate and paralyzed using 2 mM levamisole. Whole-worm images were captured using a Nikon SMZ 1500 dissecting microscope (Tokyo, Japan) with an Allied Vision Tech Stingray F-145 B Firewire Camera using Vimba image acquisition software (Allied Vision, Exton, PA, USA).

### Degeneration of dopamine neurons and quantification of dendritic blebs

The degeneration of dopamine neurons was monitored by expressing GFP specifically in dopamine neurons under the *dat-1* dopamine transporter promoter. It was previously shown that treatments that cause a loss of GFP-positive neurons cause a corresponding loss of dopamine neuron cell bodies.^63^ Synchronized P_dat-1_:GFP worms were plated onto 25 μM FUdR agar plates to be observed at increasing ages. At each time point 10–15 worms were mounted onto a 1% agar pad on a glass slide, immobilized using 2 mM levamisole, and enclosed with a coverslip. Imaging of immobilized animals was carried out with an Axioplan 2 inverted fluorescence microscope (Zeiss, Oberkochen, Germany). Quantification of dopamine neurons and dendritic blebs was done in a blinded manner.

### Measurement of aggregation

#### Localized aggregate counts

Worms were mounted onto slides as described above. Fluorescent punctae were then manually counted in a defined region centered around the vulva using a ×63 objective on a Zeiss Axioplan 2 compound microscope (Zeiss, Oberkochen, Germany).

#### Whole-worm aggregate measurements

Whole-worm images were captured as described for lipofuscin measurements. Punctae, defined as clusters of at least 3 pixels whose intensity were at least 1 standard deviation above the background intensity, were quantified using MATLAB.

## References

[bib1] Polymeropoulos, M. H. et al. Mutation in the alpha-synuclein gene identified in families with Parkinson's disease. Science 276: 2045–2047 (1997). 9197268 10.1126/science.276.5321.2045

[bib2] Singleton, A. B. et al. alpha-Synuclein locus triplication causes Parkinson's disease. Science 302: 841 (2003). 14593171 10.1126/science.1090278

[bib3] Spillantini, M. G. , Crowther, R. A. , Jakes, R. , Hasegawa, M. & Goedert, M. alpha-Synuclein in filamentous inclusions of Lewy bodies from Parkinson's disease and dementia with lewy bodies. Proc. Natl Acad. Sci. USA 95: 6469–6473 (1998). 9600990 10.1073/pnas.95.11.6469 PMC27806

[bib4] Zimprich, A. et al. Mutations in LRRK2 cause autosomal-dominant parkinsonism with pleomorphic pathology. Neuron 44: 601–607 (2004). 15541309 10.1016/j.neuron.2004.11.005

[bib5] Paisan-Ruiz, C. et al. Cloning of the gene containing mutations that cause PARK8-linked Parkinson's disease. Neuron 44: 595–600 (2004). 15541308 10.1016/j.neuron.2004.10.023

[bib6] Driver, J. A. , Logroscino, G. , Gaziano, J. M. & Kurth, T. Incidence and remaining lifetime risk of Parkinson disease in advanced age. Neurology 72: 432–438 (2009). 19188574 10.1212/01.wnl.0000341769.50075.bb PMC2676726

[bib7] Tan, J. M. , Wong, E. S. & Lim, K. L. Protein misfolding and aggregation in Parkinson's disease. Antioxid. Redox Signal. 11: 2119–2134 (2009). 19243238 10.1089/ars.2009.2490

[bib8] Zhou, C. , Huang, Y. & Przedborski, S. Oxidative stress in Parkinson's disease: a mechanism of pathogenic and therapeutic significance. Ann. N. Y. Acad. Sci. 1147, 93–104 (2008). 19076434 10.1196/annals.1427.023 PMC2745097

[bib9] Henchcliffe, C. & Beal, M. F. Mitochondrial biology and oxidative stress in Parkinson disease pathogenesis. Nat. Clin. Pract. Neurol. 4: 600–609 (2008). 18978800 10.1038/ncpneuro0924

[bib10] Cook, C. & Petrucelli, L. A critical evaluation of the ubiquitin-proteasome system in Parkinson's disease. Biochim. Biophys. Acta 1792: 664–675 (2009). 19419700 10.1016/j.bbadis.2009.01.012 PMC2828612

[bib11] Pan, T. , Kondo, S. , Le, W. & Jankovic, J. The role of autophagy-lysosome pathway in neurodegeneration associated with Parkinson's disease. Brain 131: 1969–1978 (2008). 18187492 10.1093/brain/awm318

[bib12] Kenyon, C. , Chang, J. , Gensch, E. , Rudner, A. & Tabtiang, R. A. C. elegans mutant that lives twice as long as wild type. Nature 366: 461–464. (1993). 8247153 10.1038/366461a0

[bib13] Wong, A. , Boutis, P. & Hekimi, S. Mutations in the clk-1 gene of Caenorhabditis elegans affect developmental and behavioral timing. Genetics 139: 1247–1259 (1995). 7768437 10.1093/genetics/139.3.1247 PMC1206454

[bib14] Friedman, D. B. & Johnson, T. E. A mutation in the age-1 gene in Caenorhabditis elegans lengthens life and reduces hermaphrodite fertility. Genetics 118: 75–86 (1988). 8608934 10.1093/genetics/118.1.75 PMC1203268

[bib15] Tacutu, R. et al. Human Ageing Genomic Resources: Integrated databases and tools for the biology and genetics of ageing. Nucleic Acids Res. 41 (2012). 10.1093/nar/gks1155 PMC3531213 23193293

[bib16] Holzenberger, M. et al. IGF-1 receptor regulates lifespan and resistance to oxidative stress in mice. Nature 421: 182–187 (2003). 12483226 10.1038/nature01298

[bib17] Suh, Y. et al. Functionally significant insulin-like growth factor I receptor mutations in centenarians. Proc. Natl Acad. Sci. USA 105: 3438–3442 (2008). 18316725 10.1073/pnas.0705467105 PMC2265137

[bib18] Lakso, M. et al. Dopaminergic neuronal loss and motor deficits in Caenorhabditis elegans overexpressing human alpha-synuclein. J. Neurochem. 86: 165–172 (2003). 12807436 10.1046/j.1471-4159.2003.01809.x

[bib19] Saha, S. , Liu-Yesucevitz, L. & Wolozin, B. Regulation of autophagy by LRRK2 in Caenorhabditis elegans. Neurodegener. DIseases 13: 110–113 (2014). 10.1159/000355654 PMC3946265 24192129

[bib20] Kuwahara, T. et al. Familial Parkinson mutant alpha-synuclein causes dopamine neuron dysfunction in transgenic Caenorhabditis elegans. J. Biol. Chem. 281: 334–340 (2006). 16260788 10.1074/jbc.M504860200

[bib21] Yao, C. et al. LRRK2-mediated neurodegeneration and dysfunction of dopaminergic neurons in a Caenorhabditis elegans model of Parkinson's disease. Neurobiol. Dis. 40: 73–81 (2010). 20382224 10.1016/j.nbd.2010.04.002 PMC2926296

[bib22] Ved, R. et al. Similar patterns of mitochondrial vulnerability and rescue induced by genetic modification of alpha-synuclein, parkin, and DJ-1 in Caenorhabditis elegans. J. Biol. Chem. 280: 42655–42668 (2005). 16239214 10.1074/jbc.M505910200 PMC3910375

[bib23] Samann, J. et al. Caenorhabditits elegans LRK-1 and PINK-1 act antagonistically in stress response and neurite outgrowth. J. Biol. Chem. 284: 16482–16491 (2009). 19251702 10.1074/jbc.M808255200 PMC2713553

[bib24] van Ham, T. J. et al. C. elegans model identifies genetic modifiers of alpha-synuclein inclusion formation during aging. PLoS Genet. 4: e1000027 (2008). 18369446 10.1371/journal.pgen.1000027 PMC2265412

[bib25] Sawin, E. R. , Ranganathan, R. & Horvitz, H. R. C. elegans locomotory rate is modulated by the environment through a dopaminergic pathway and by experience through a serotonergic pathway. Neuron 26: 619–631 (2000). 10896158 10.1016/s0896-6273(00)81199-x

[bib26] Lee, J. , Jee, C. & McIntire, S. L. Ethanol preference in C. elegans. Genes Brain Behav. 8: 578–585 (2009). 19614755 10.1111/j.1601-183X.2009.00513.x PMC2880621

[bib27] Hills, T. , Brockie, P. J. & Maricq, A. V. Dopamine and glutamate control area-restricted search behavior in Caenorhabditis elegans. J. Neurosci. 24: 1217–1225 (2004). 14762140 10.1523/JNEUROSCI.1569-03.2004 PMC6793574

[bib28] Lints, R. & Emmons, S. W. cat-2 encodes a putative tyrosine hydroxylase that is essential for dopamine biosynthesis. Worm Breeder's Gazette 15: 21 (1998).

[bib29] Tucci, M. L. , Harrington, A. J. , Caldwell, G. A. & Caldwell, K. A. Modeling dopamine neuron degeneration in Caenorhabditis elegans. Methods Mol. Biol. 793, 129–148 (2011). 21913098 10.1007/978-1-61779-328-8_9

[bib30] Lithgow, G. J. , White, T. M. , Melov, S. & Johnson, T. E. Thermotolerance and extended life-span conferred by single-gene mutations and induced by thermal stress. Proc. Natl Acad. Sci. USA 92: 7540–7544 (1995). 7638227 10.1073/pnas.92.16.7540 PMC41375

[bib31] Honda, Y. & Honda, S. The daf-2 gene network for longevity regulates oxidative stress resistance and Mn-superoxide dismutase gene expression in Caenorhabditis elegans. FASEB J. 13: 1385–1393 (1999). 10428762

[bib32] Lamitina, S. T. & Strange, K. Transcriptional targets of DAF-16 insulin signaling pathway protect C. elegans from extreme hypertonic stress. Am. J. Physiol. Cell Physiol. 288, C467–C474 (2005). 15496475 10.1152/ajpcell.00451.2004

[bib33] Collier, T. J. , Kanaan, N. M. & Kordower, J. H. Ageing as a primary risk factor for Parkinson's disease: evidence from studies of non-human primates. Nat. Rev. Neurosci. 12: 359–366 (2011). 21587290 10.1038/nrn3039 PMC3387674

[bib34] Levy, G. The relationship of Parkinson disease with aging. Arch. Neurol. 64: 1242–1246 (2007). 17846263 10.1001/archneur.64.9.1242

[bib35] Rodriguez, M. , Rodriguez-Sabate, C. , Morales, I. , Sanchez, A. & Sabate, M. Parkinson's disease as a result of aging. Aging Cell 14: 293–308 (2015). 25677794 10.1111/acel.12312 PMC4406659

[bib36] Li, W. et al. Stabilization of alpha-synuclein protein with aging and familial parkinson's disease-linked A53T mutation. J. Neurosci. 24: 7400–7409 (2004). 15317865 10.1523/JNEUROSCI.1370-04.2004 PMC6729772

[bib37] Verstraeten, A. , Theuns, J. & Van Broeckhoven, C. Progress in unraveling the genetic etiology of Parkinson disease in a genomic era. Trends Genet. 31: 140–149 (2015). 25703649 10.1016/j.tig.2015.01.004

[bib38] Lee, S. J. & Kenyon, C. Regulation of the longevity response to temperature by thermosensory neurons in Caenorhabditis elegans. Curr. Biol. 19: 715–722 (2009). 19375320 10.1016/j.cub.2009.03.041 PMC2868911

[bib39] Weinshenker, D. , Garriga, G. & Thomas, J. H. Genetic and pharmacological analysis of neurotransmitters controlling egg laying in C. elegans. J. Neurosci. 15: 6975–6985 (1995). 7472454 10.1523/JNEUROSCI.15-10-06975.1995 PMC6577982

[bib40] Kenyon, C. J. The genetics of ageing. Nature 464: 504–512 (2010). 20336132 10.1038/nature08980

[bib41] Knight, A. L. et al. The glycolytic enzyme, GPI, is a functionally conserved modifier of dopaminergic neurodegeneration in Parkinson's models. Cell Metab. 20: 145–157 (2014). 24882066 10.1016/j.cmet.2014.04.017 PMC4097176

[bib42] Wood, J. G. et al. Sirtuin activators mimic caloric restriction and delay ageing in metazoans. Nature 430: 686–689 (2004). 15254550 10.1038/nature02789

[bib43] Jin, F. , Wu, Q. , Lu, Y. F. , Gong, Q. H. & Shi, J. S. Neuroprotective effect of resveratrol on 6-OHDA-induced Parkinson's disease in rats. Eur. J. Pharmacol. 600: 78–82 (2008). 18940189 10.1016/j.ejphar.2008.10.005

[bib44] Harrison, D. E. et al. Rapamycin fed late in life extends lifespan in genetically heterogeneous mice. Nature 460: 392–395 (2009). 19587680 10.1038/nature08221 PMC2786175

[bib45] Malagelada, C. , Jin, Z. H. , Jackson-Lewis, V. , Przedborski, S. & Greene, L. A. Rapamycin protects against neuron death in in vitro and in vivo models of Parkinson's disease. J. Neurosci. 30: 1166–1175 (2010). 20089925 10.1523/JNEUROSCI.3944-09.2010 PMC2880868

[bib46] Anderson, R. M. , Shanmuganayagam, D. & Weindruch, R. Caloric restriction and aging: studies in mice and monkeys. Toxicol. Pathol. (2009); 37: 47–51. 19075044 10.1177/0192623308329476 PMC3734859

[bib47] Duan, W. & Mattson, M. P. Dietary restriction and 2-deoxyglucose administration improve behavioral outcome and reduce degeneration of dopaminergic neurons in models of Parkinson's disease. J. Neurosci. Res. 57: 195–206 (1999). 10398297 10.1002/(SICI)1097-4547(19990715)57:2<195::AID-JNR5>3.0.CO;2-P

[bib48] Maswood, N. et al. Caloric restriction increases neurotrophic factor levels and attenuates neurochemical and behavioral deficits in a primate model of Parkinson's disease. Proc. Natl Acad. Sci. USA 101: 18171–18176 (2004). 15604149 10.1073/pnas.0405831102 PMC539733

[bib49] Onken, B. & Driscoll, M. Metformin induces a dietary restriction-like state and the oxidative stress response to extend C. elegans Healthspan via AMPK, LKB1, and SKN-1. PLoS ONE 5: e8758 (2010). 20090912 10.1371/journal.pone.0008758 PMC2807458

[bib50] Martin-Montalvo, A. et al. Metformin improves healthspan and lifespan in mice. Nat. Commun. 4, 2192 (2013). 23900241 10.1038/ncomms3192 PMC3736576

[bib51] Patil, S. P. , Jain, P. D. , Ghumatkar, P. J. , Tambe, R. & Sathaye, S. Neuroprotective effect of metformin in MPTP-induced Parkinson's disease in mice. Neuroscience 277, 747–754 (2014). 25108167 10.1016/j.neuroscience.2014.07.046

[bib52] Bluher, M. , Kahn, B. B. & Kahn, C. R. Extended longevity in mice lacking the insulin receptor in adipose tissue. Science 299: 572–574 (2003). 12543978 10.1126/science.1078223

[bib53] Taguchi, A. , Wartschow, L. M. & White, M. F. Brain IRS2 signaling coordinates life span and nutrient homeostasis. Science 317: 369–372 (2007). 17641201 10.1126/science.1142179

[bib54] Coschigano, K. T. , Clemmons, D. , Bellush, L. L. & Kopchick, J. J. Assessment of growth parameters and life span of GHR/BP gene-disrupted mice. Endocrinology 141: 2608–2613 (2000). 10875265 10.1210/endo.141.7.7586

[bib55] Flurkey, K. , Papaconstantinou, J. , Miller, R. A. & Harrison, D. E. Lifespan extension and delayed immune and collagen aging in mutant mice with defects in growth hormone production. Proc. Natl Acad. Sci. USA 98: 6736–6741 (2001). 11371619 10.1073/pnas.111158898 PMC34422

[bib56] Brown-Borg, H. M. , Borg, K. E. , Meliska, C. J. & Bartke, A. Dwarf mice and the ageing process. Nature 384: 33 (1996). 10.1038/384033a0 8900272

[bib57] Lorenzini, A. et al. Mice producing reduced levels of insulin-like growth factor type 1 display an increase in maximum, but not mean, life span. J. Gerontol. A Biol. Sci. Med. Sci. 69: 410–419 (2014). 23873963 10.1093/gerona/glt108 PMC3968822

[bib58] Wolkow, C. A. , Kimura, K. D. , Lee, M. S. & Ruvkun, G. Regulation of C. elegans life-span by insulinlike signaling in the nervous system. Science 290: 147–150 (2000). 11021802 10.1126/science.290.5489.147

[bib59] Nadjar, A. et al. IGF-1 signaling reduces neuro-inflammatory response and sensitivity of neurons to MPTP. Neurobiol. Aging 30: 2021–2030 (2009). 18394756 10.1016/j.neurobiolaging.2008.02.009

[bib60] Cohen, E. et al. Reduced IGF-1 signaling delays age-associated proteotoxicity in mice. Cell 139: 1157–1169 (2009). 20005808 10.1016/j.cell.2009.11.014 PMC3017511

[bib61] Sadagurski, M. et al. IRS2 increases mitochondrial dysfunction and oxidative stress in a mouse model of Huntington disease. J. Clin. Invest. 121: 4070–4081 (2011). 21926467 10.1172/JCI46305 PMC3195462

[bib62] Van Raamsdonk, J. M. & Hekimi, S. FUdR causes a twofold increase in the lifespan of the mitochondrial mutant gas-1. Mech. Ageing Dev. 132: 519–521 (2011). 21893079 10.1016/j.mad.2011.08.006 PMC4074524

[bib63] Nass, R. , Hall, D. H. , Miller, D. M. 3rd & Blakely, R. D. Neurotoxin-induced degeneration of dopamine neurons in Caenorhabditis elegans. Proc. Natl Acad. Sci. USA 99: 3264–3269 (2002). 11867711 10.1073/pnas.042497999 PMC122507

