# Supplementary material for: Delaying aging is neuroprotective in Parkinson’s disease: a genetic analysis in C. elegans models
Source: NPJ Parkinsons Dis. 2015 Nov 19;1:15022–. doi: 10.1038/npjparkd.2015.22 (PMC5516561; doi:10.1038/npjparkd.2015.22)
Supplement: Supplementary Information [file npjparkd201522-s1.pdf]

Delaying aging is neuroprotective in Parkinson's disease: a genetic analysis in *C. elegans* models

Jason F. Cooper*[1], Dylan J. Dues*[1], Katie K. Spielbauer[1], Emily Machiela[1], Megan M. Senchuk[1], Jeremy M. Van Raamsdonk[1,2,3]

Center for Neurodegenerative Science, Van Andel Research Institute, Grand Rapids MI 49503
Department of Translational Science and Molecular Medicine, Michigan State University, Grand Rapids MI, 49503
Department of Genetics, Michigan State University, East Lansing MI 48824

Supplementary Table S1. Comparison of phenotypes caused by wild-type and mutant forms of LRRK2 and α-synuclein. The expression of both wild-type or mutant LRRK2 or α-synuclein causes phenotypic deficits in worms. Worms expressing the mutant form of the protein exhibited more severe deficits than worms expressing the wild-type form.

**Phenotype**	**LRRK2(WT)**	**LRRK2(G2019S)**	**α-syn(WT)**	**α-syn(A53T)**
Our study				
Basal slowing	Deficit (-76%)	Deficit (-90%)	No deficit	Deficit (-115%)
Ethanol avoidance	Deficit (-60%)	Deficit (-102%)	Deficit (-70%)	Deficit (-97%)
Area-restricted searching	No deficit	Deficit (-55%)	Deficit (-48%)	Deficit (-74%)
Heat stress resistance	Deficit (-43%)	Deficit (-38%)	Deficit (-67%)	Deficit (-70%)
Fertility (brood size)	No Deficit	Deficit (-48%)	Not done	No deficit
Yao et al., 2010				
Loss of dopamine neurons	Deficit (*-47%*)	Deficit (*-60%*)		
Basal slowing	Deficit (*-45%*)	Deficit (*-91%*)		
Dopamine levels	Deficit (*-47%*)	Deficit (*-74%*)		
Kuwahara et al., 2006				
Loss of neurites			Deficit (*-30%*)	Deficit (*-80%*)
Basal slowing			No deficit	Deficit (*-43%*)
Dopamine levels			Deficit (*-40%*)	Deficit (*-70%*)

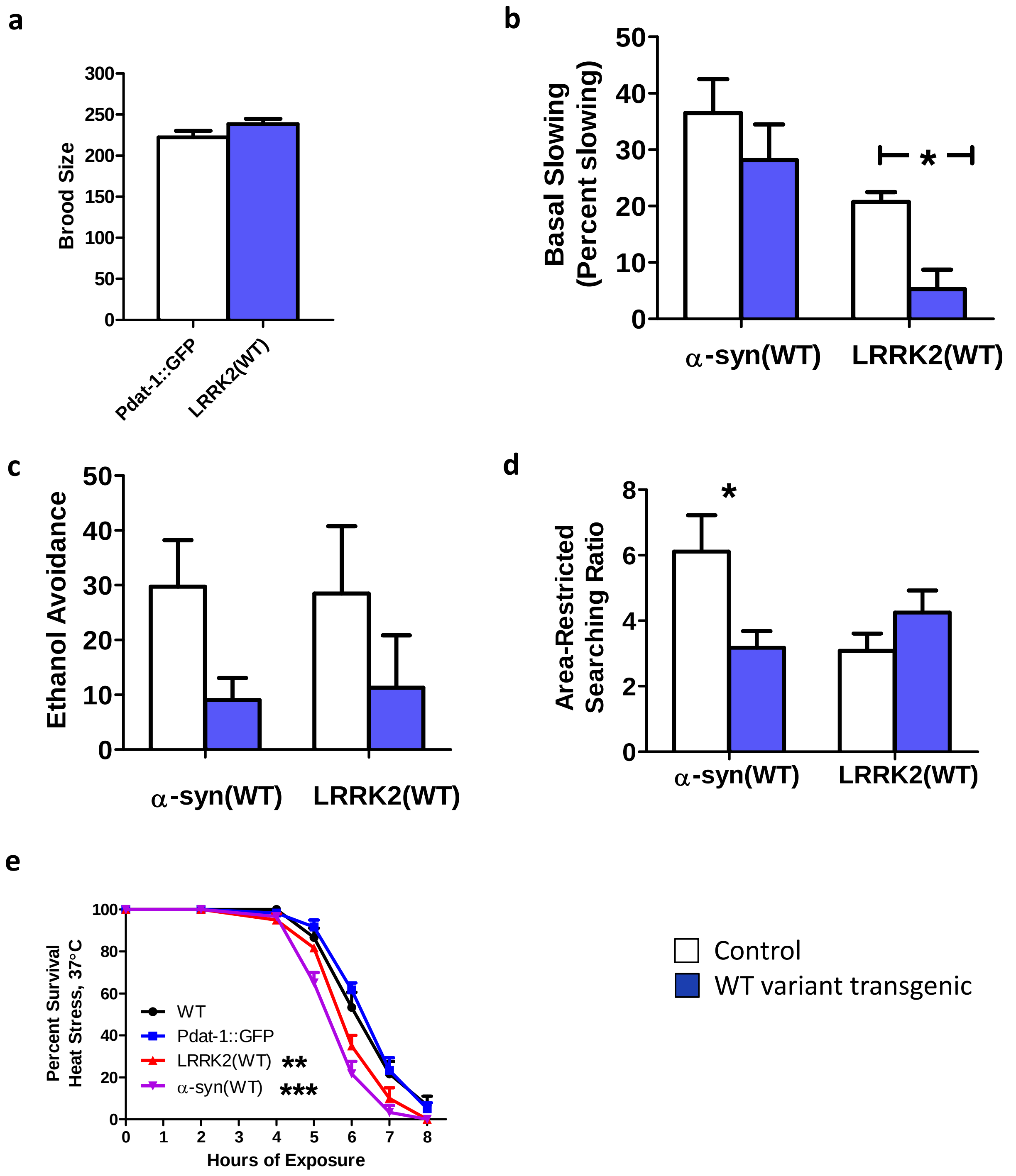

Supplementary Figure S1. Expression of wild-type α-synuclein or LRRK2 in dopamine neurons results in mild phenotypic abnormalities. To determine the extent to which deficits observed in worm models of Parkinson's disease expressing human mutant α-syn or LRRK2 are also present when the wild-type protein is overexpressed in dopamine neurons, we characterized Pdat-1::α-syn(WT) and Pdat-1::LRRK2(WT),Pdat-1::GFP worms. **a.** Expression of wild-type LRRK2 did not decrease brood size. **b.** α-syn(WT) worms have normal basal slowing, while LRRK2(WT) worms exhibit impaired basal slowing. **c.** There was a trend towards decreased ethanol avoidance in both α-syn(WT) and LRRK2(WT) worms. **d.** α-syn(WT) worms exhibit a deficit in area-restricted searching while LRRK2(WT) worms do not. **e.** The expression of WT α-syn or LRRK2 in dopamine neurons increases sensitivity to heat stress. Error bars indicate SEM. *p<0.05, **p<0.01, ***p<0.001.

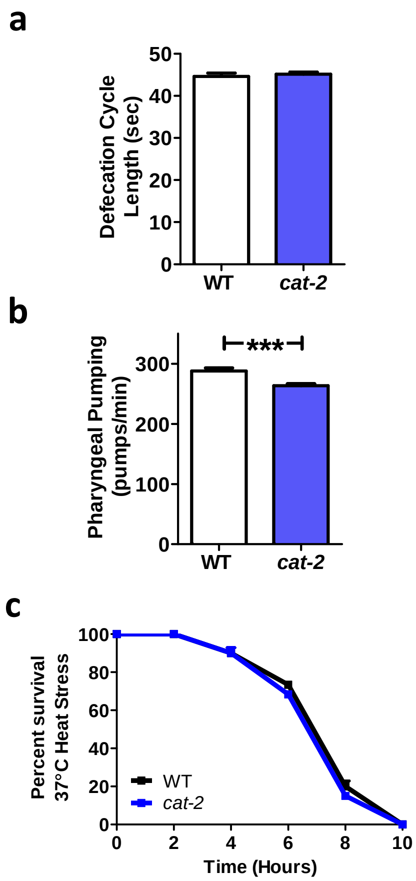

Supplementary Figure S2. Dopamine dependency of behavioral phenotypes. *cat-2* worms have a mutation in the gene encoding tyrosine hydroxylase, which catalyzes the rate limiting step in the synthesis of dopamine. As a result, *cat-2* mutants have depleted levels of dopamine. *cat-2* worms exhibit a normal defecation cycle length **(a)**, normal sensitivity to heat stress **(c)** but show a decreased rate of pharyngeal pumping **(b)**. This suggests that pharyngeal pumping may be a dopamine-dependent behavior while defecation cycle length and resistance to heat stress appear to be dopamine independent. Error bars indicate SEM. ***$p<0.001$.

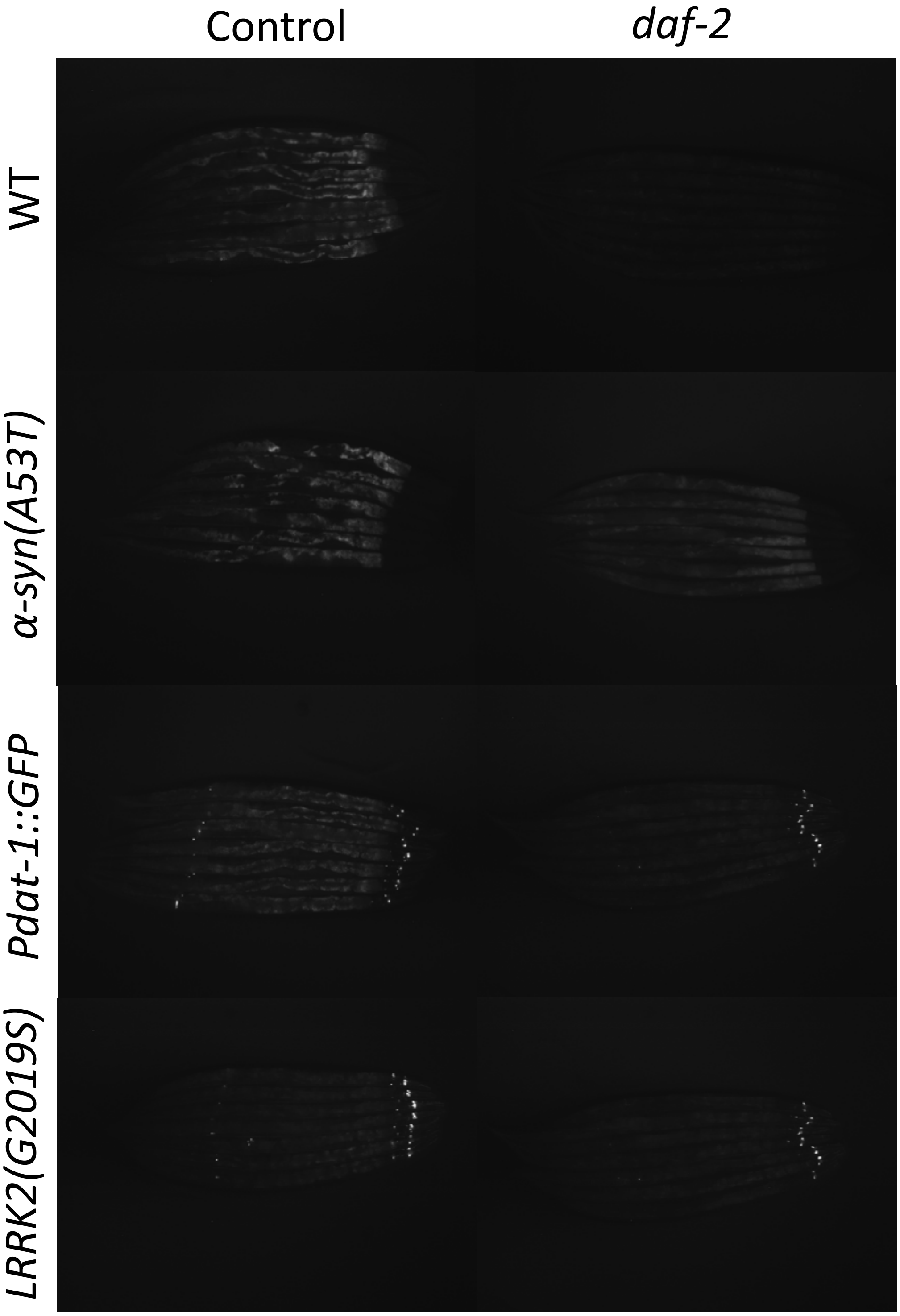

Supplementary Figure S3. *daf-2* mutation decreases lipofuscin accumulation in Parkinson's disease worms (day 6). Transgenic worm models of PD expressing human mutant α-synuclein (A53T mutation) or LRRK2 (G2019S mutation) were crossed to long-lived *daf-2* mutants. The increased lifespan caused by the *daf-2* mutation decreased lipofuscin accumulation on day 6 of adulthood in both the α-synuclein (*Pdat-1::α-syn(A53T)*) and LRRK2 (*Pdat-1::LRRK2(G2019S);Pdat-1::GFP*) models of PD.

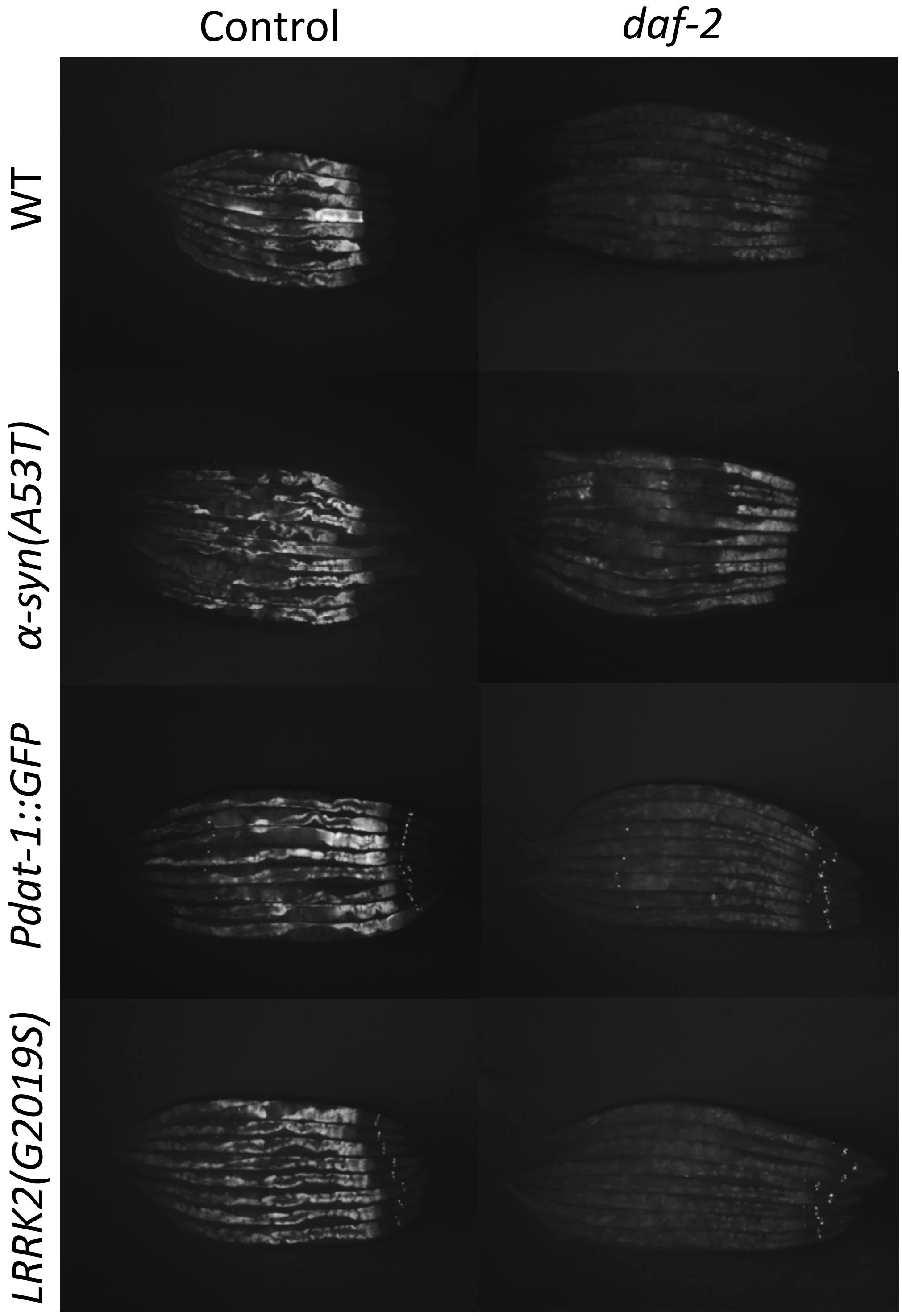

Supplementary Figure S4. *daf-2* mutation decreases lipofuscin accumulation in Parkinson's disease worms (day 9). Transgenic worm models of PD expressing human mutant α-synuclein (A53T mutation) or LRRK2 (G2019S mutation) were crossed to long-lived *daf-2* mutants. The increased lifespan caused by the *daf-2* mutation decreased lipofuscin accumulation in both the α-synuclein (*Pdat-1::α-syn(A53T)*) and LRRK2 (*Pdat-1::LRRK2(G2019S);Pdat-1::GFP*) models of PD on day 9 of adulthood.

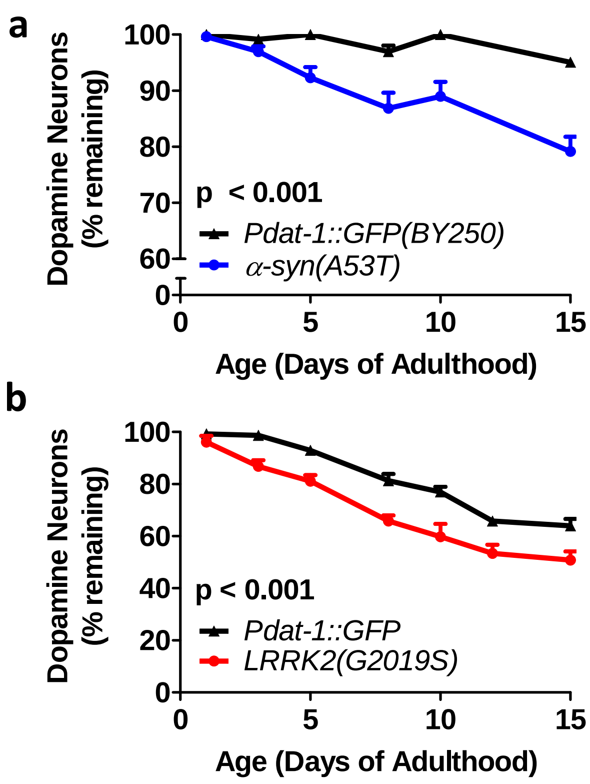

Supplementary Figure S5. Worm models of Parkinson's disease have accelerated loss of dopamine neurons. Dopamine neurons were identified by expressing GFP under the *dat-1* dopamine transporter promoter. While wild-type worms exhibit a loss of GFP-positive dopamine neurons with age, this loss is accelerated in both α-synuclein(A53T) (**a**) and LRRK2(G2019S) (**b**) worm models of PD. Error bars indicate SEM.

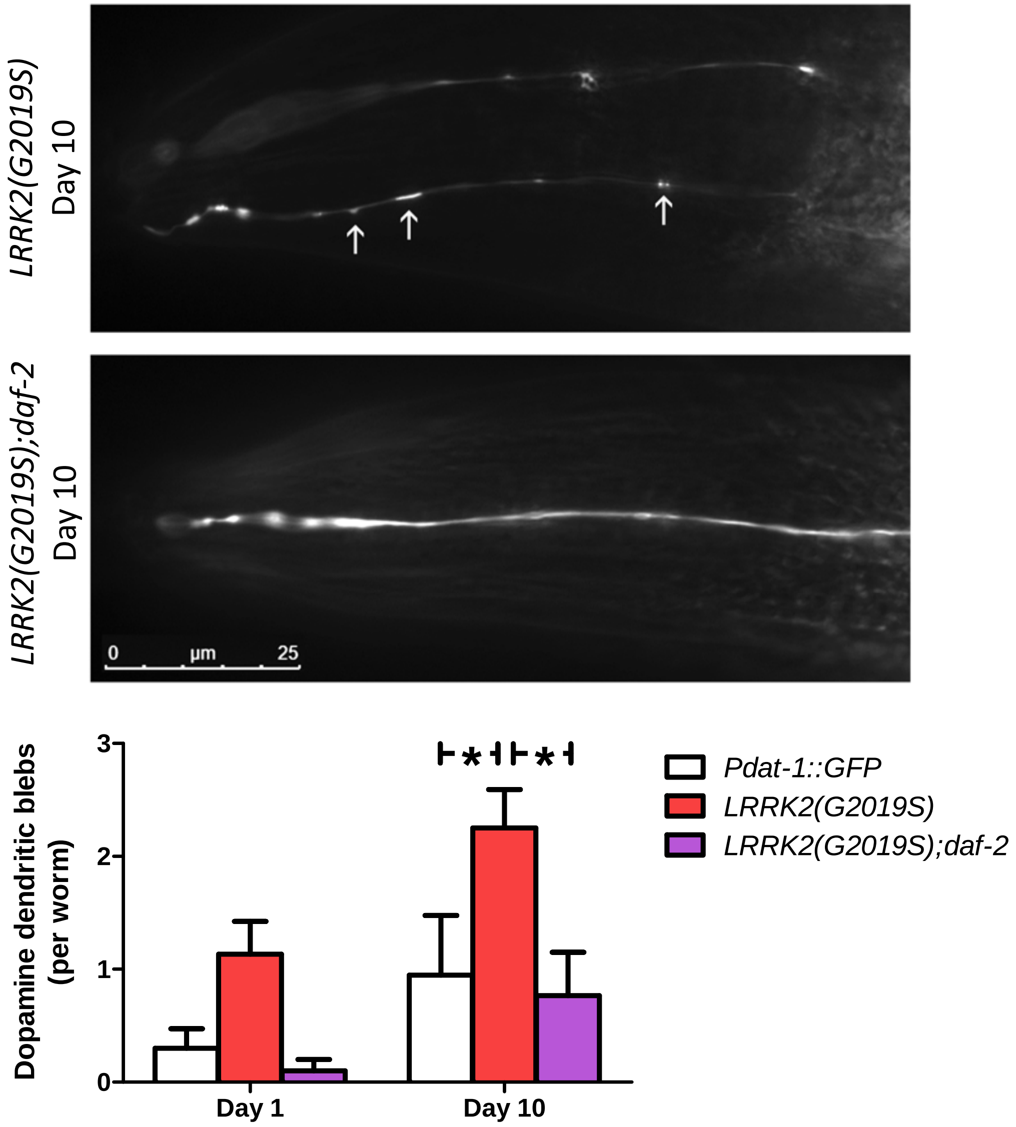

Supplementary Figure S6. Expression of human mutant LRRK2 with G2019S mutation causes neuronal blebs in dendrites that increase with age and are reduced by *daf-2* mutation. The degeneration of dendrites was monitored by quantifying the number of blebs present at day 1 and day 10. Examples of blebs are indicated by the arrows. Blebs were found to increase with age in all strains examined. More blebs were observed in LRRK2(G2019S) mutant worms than control. Delaying aging through *daf-2* mutation reduced the number of blebs present. Error bars indicate SEM. *p<0.05.

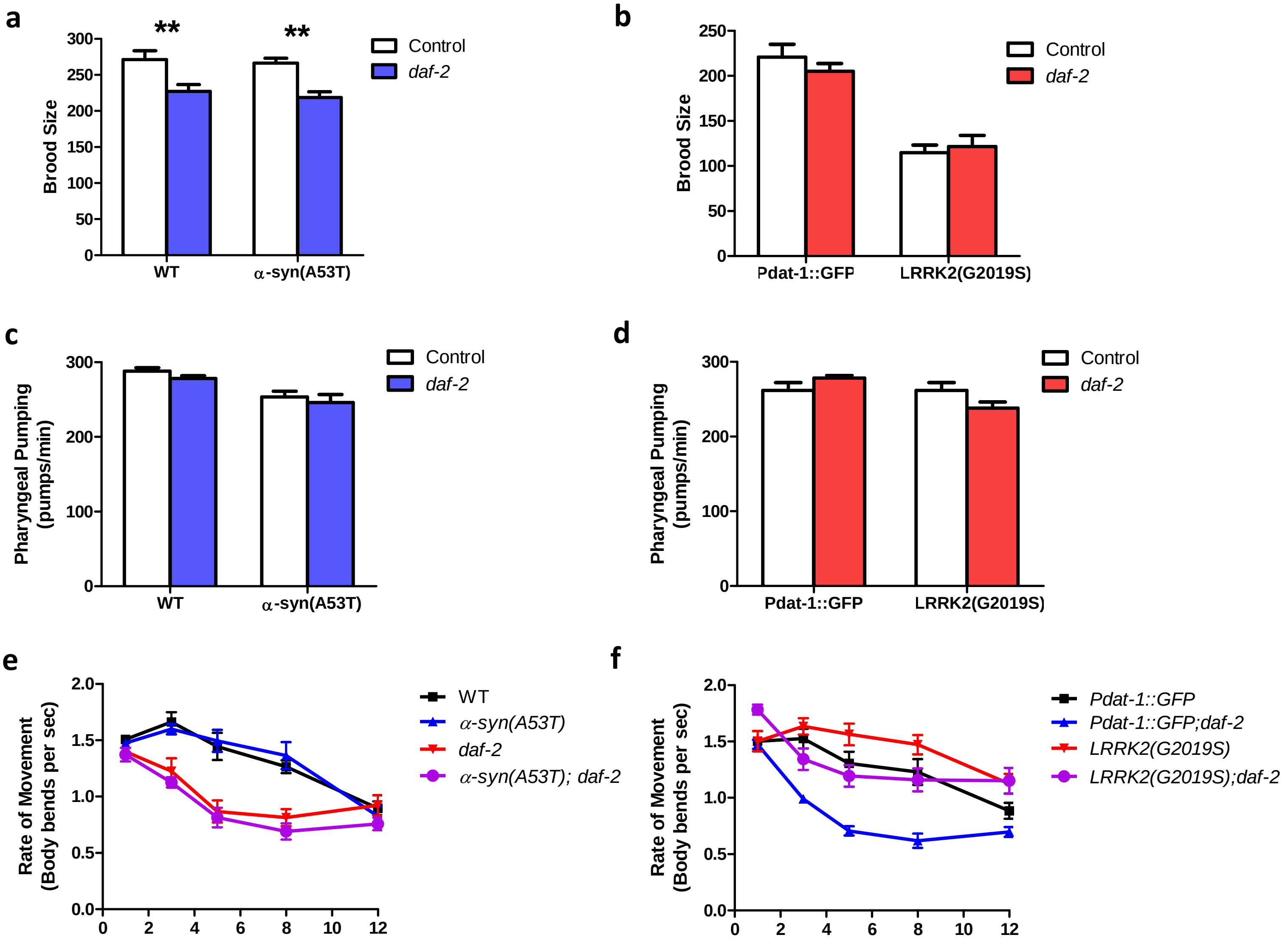

Supplementary Figure S7. Delaying aging through mutation of *daf-2* does not improve brood size, pharyngeal pumping rate or rate of movement in worm models of Parkinson's disease. a. *daf-2* mutant worms exhibit decreased fertility and reduce the brood size in α-syn(A53T) PD mutant worms. **b.** While the *daf-2* mutation did not improve the low brood size in LRRK2(G2019S) worms, there was no further decrease in brood size resulting from the *daf-2* mutations. **c,d.** The *daf-2* mutation had no significant effect on pharyngeal pumping in WT or PD mutant worms. **e.** The *daf-2* mutation resulted in decreased movement in a WT or α-syn(A53T) mutant background. **f.** Interestingly, the LRRK2(G2019S) mutation increases the rate of movement in *daf-2* worms throughout lifespan. Error bars indicate SEM.

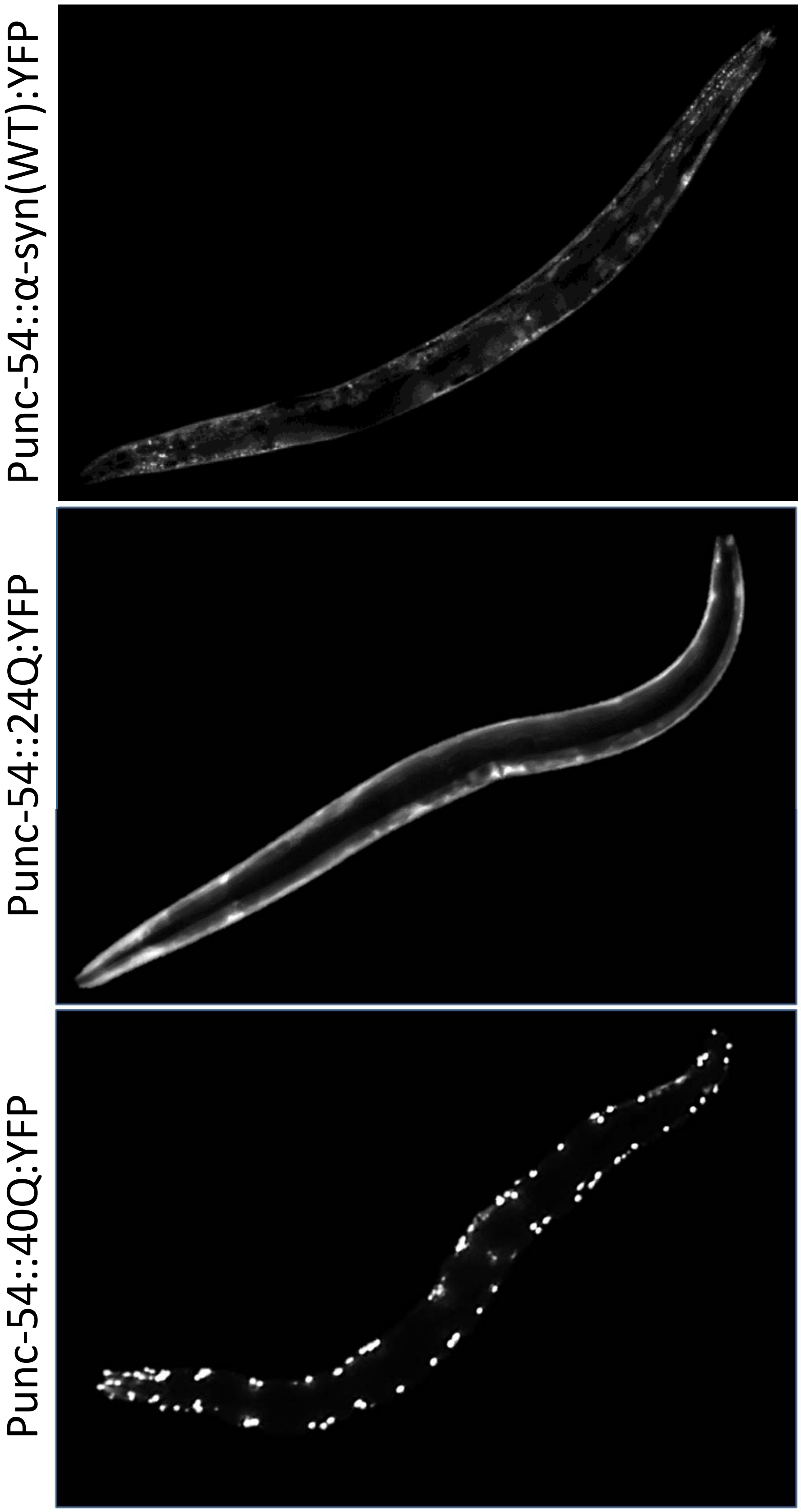

Supplementary Figure S8. Worms expressing α-synuclein in body wall muscle exhibit the formation of small aggregates. For comparison worms expressing polyglutamine linked to YFP in unaffected range (24Q, top) and disease range (40Q, middle) are shown. Unlike the disease-length polyglutamine protein that forms large aggregates and exhibits no diffuse expression in the body wall, α-synuclein primarily forms small aggregates and always exhibits some diffuse expression.

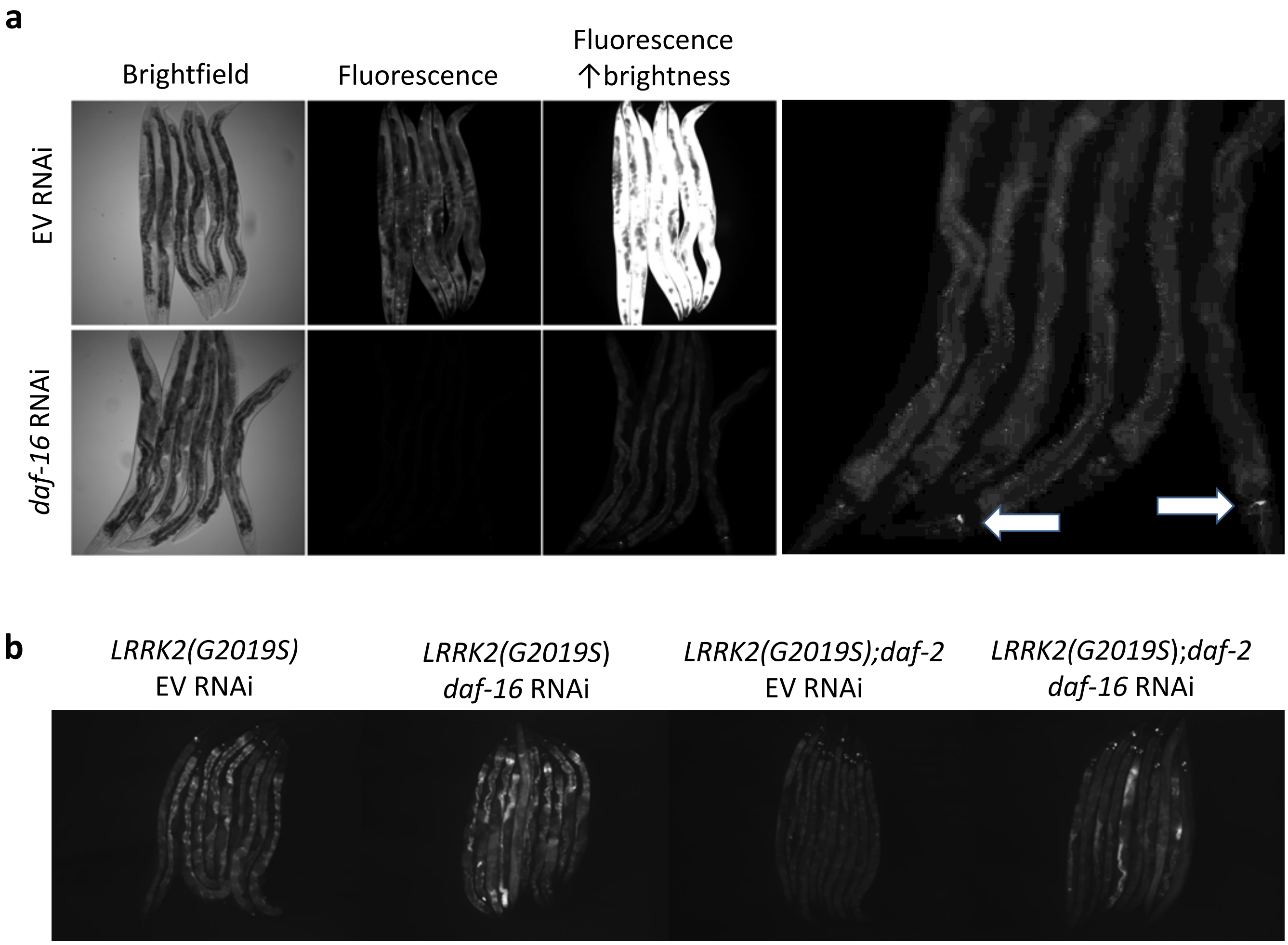

Supplementary Figure S9. Treatment with *daf-16* RNAi decreases expression of *daf-16* and delays accumulation of lipofuscin. a. To test the effectiveness of *daf-16* RNAi, *Pdaf-16::daf-16:GFP* worms were treated with RNAi bacteria and imaged. *daf-16* RNAi markedly reduced the expression of DAF-16:GFP. Increasing brightness and contrast revealed that there was still some DAF-16:GFP present in neurons. Neurons are known to have decreased RNAi efficiency. Note that the intestinal fluorescence observed is lipofuscin. **b.** Lipofuscin measurements were used as a biomarker of aging. The *daf-2* mutation decreases lipofuscin accumulation and this was partially prevented by *daf-16* RNAi. This is consistent with the fact that *daf-16* RNAi decreases *LRRK2(G2019S);daf-2* lifespan.
